# Historical red-lining is associated with fossil fuel power plant siting and present-day inequalities in air pollutant emissions

**DOI:** 10.1038/s41560-022-01162-y

**Published:** 2022-12-15

**Authors:** Lara J. Cushing, Shiwen Li, Benjamin B. Steiger, Joan A. Casey

**Affiliations:** 1 Department of Environmental Health Sciences, University of California Los Angeles, Los Angeles, CA, USA.; 2 Department of Epidemiology, University of California Los Angeles, Los Angeles, CA, USA.; 3 Department of Environmental Health Sciences, Columbia Mailman School of Public Health, New York, NY, USA.

## Abstract

Stationary sources of air pollution are disproportionately located in communities of colour, but the causes for this disparity are unclear. Here we assess whether racialized appraisals of investment risk (‘red-lining’) undertaken by the US federal Home Owners’ Loan Corporation in the 1930s influenced the subsequent siting of fossil fuel power plants. Across 8,871 neighbourhoods in 196 US urban areas, we observed a stepwise correlation between risk grade, number of power plants and cumulative quantity of power plant emissions upwind and within 5 km. Controlling for pre-existing power plants, neighbourhoods deemed ‘hazardous’ (D grade, ‘red-lined’) had a higher likelihood of a fossil fuel power plant being sited between 1940 and 1969 (72%), 1970 and 1999 (20%) and 2000 and 2019 (31%), and higher average present-day emissions of nitrous oxides (82%), sulfur dioxide (38%) and fine particulate matter (63%) compared with ‘declining’ ( C- graded) neighbourhoods. Our results suggest racism in the housing market contributed to inequalities in present-day power plant emissions burdens.

Ambient air pollution is estimated to cause over 100,000 premature deaths in the United States each year^1^. Black, Hispanic and Asian communities face higher exposures to hazardous and criteria ambient air pollutants^2–5^ and are more likely to live near polluting industries, even when accounting for differences in income and educational attainment^6^. Fossil fuel power plants contribute to local and regional air pollution through the emission of particulate matter (PM), nitrogen oxides (NO_*x*_) and—in the case of coal-fired power plants—sulfur oxides and mercury. The burden of PM emissions from coal-fired power plants is greater for Americans of colour than White Americans, and emissions reductions due to recent coal-fired power plant retirements appear to have disproportionately benefited White Americans^7^. However, the processes and historical factors that led to these present-day inequalities are poorly understood, and the forms of structural racism contributing to racial disparities in air pollution exposures remain under-explored.

In the wake of the Great Depression, the newly formed US federal Home Owners’ Loan Corporation (HOLC) and Federal Housing Administration undertook widespread neighbourhood appraisals as part of an effort to facilitate access to affordable home mortgages and prevent foreclosures. HOLC Residential Security Maps created in the 1930s in consultation with the local developers, financial and real estate professionals ranked neighbourhoods according to perceived lending risk and assigned grades that typically ranged from A (‘best’) to D (‘hazardous’)^8^. The practice became known as ‘red-lining’ because of the red colour given to D-graded neighbourhoods with the highest perceived investment risk. The maps reflected prevailing racist beliefs in the market value of integrated neighbourhoods such that communities with Black, East Asian, Filipino and foreign-born residents received poorer grades, while A-grade neighbourhoods appear to have been almost exclusively made up of White, US-born residents^9^. Through their codification of racist ideologies about value in the residential housing market, the HOLC maps are thought to have deepened racial residential segregation already enforced via racially restrictive deed covenants, zoning, block busting and violence and contributed to the exclusion of people of colour from homeownership^10,11^. Recent scholarship suggests historical red-lining—and the racism in the housing market it reflected and codified—contributed to present-day geographic disparities in homeownership, home values, rents and income^12–14^.

The recent digitization of HOLC Residential Security Maps has enabled a growing number of studies to document a relationship between historically red-lined neighbourhoods and present-day environmental and health outcomes^15^. Red-lining has been associated with less urban vegetation^16^, higher surface temperatures^17^, higher levels of air pollution^18,19^, the siting of oil and gas wells^20^ and higher rates of preterm birth^21,22^, emergency department visits for asthma^23^ and late-stage cancer diagnoses^24^. However, studies to date have been almost exclusively cross-sectional due to a lack of historical data on these outcomes, giving little insight into the influence of red-lining on the trajectories shaping neighbourhood health and environmental inequalities over time. Also, few have attempted to control for pre-existing differences in neighbourhoods (for example, in the age of housing and socioeconomic status of residents) that pre-dated the creation of the Residential Security Maps and may partially explain differences in present-day conditions.

We investigate the relationship between HOLC grades assigned in the 1930s and the subsequent siting of fossil fuel (coal, oil or natural gas) power plants and resulting differences in the burden of present-day (2018 and 2019) power plant emissions of NO_*x*_, sulfur dioxide (SO_2_) and fine PM of 2.5 microns or less in diameter (PM_2.5_). Our primary objective was to understand whether racism in the housing market as reflected in the assignment of investment risk grades on federal HOLC Security Maps resulted in disparities in the likelihood of a power plant being sited nearby. As a second objective, we sought to assess whether these historical siting decisions led to differences in power plant emissions burdens that persist into the present day, indicating the continued relevance of historical red-lining in shaping current neighbourhood-level inequalities in proximity to environmental hazards.

When comparing D- (‘hazardous’ or ‘red-lined’) to C-graded (‘declining’) neighbourhoods, we found red-lining was associated with an increased risk of a fossil fuel plant being sited upwind and within 5 km and a higher present-day emissions burden of NO_*x*_, SO_2_ and PM_2.5_, even when accounting for pre-existing differences in proximity to power plants, socioeconomic factors and city population size. Associations with siting weakened over time but persisted into the twenty-first century, over 70 years after the first red-lining maps were drawn. Our findings suggest racism in the residential housing market has had an enduring impact on the subsequent siting of fossil fuel power plants and distribution of present-day air pollutant emissions.

## Approach

We used HOLC boundaries as our spatial unit of analysis and consider them ‘exposed’ if they were downwind of a power plant within 5 km of the HOLC boundary based on annual average wind direction. We incorporated both distance and wind direction into our metric to focus on power plants that are most likely to impact the air quality of nearby populations and because we hypothesized that wind direction may have influenced siting decisions. Sensitivity analyses in which we defined exposure only on the basis of proximity irrespective of wind direction confirmed this hypothesis in that it resulted in largely consistent but weaker effect estimates (Supplementary Table 1 provides results based on proximity, and Supplementary Table 2 provides results based on proximity and wind direction).

We assessed the siting of new fossil fuel power plants upwind and within 5 km during three time periods: pre-1940 baseline (encompassing power plants likely in the planning stages at the time of the completion of HOLC maps), 1940–1969 (post-war era), 1970–1999 (post-enactment of the Clean Air Act of 1970) and 2000–2019 (modern era including the 2005 Energy Policy Act and recent boom in domestic oil and gas production) Supplementary Fig. 1 illustrates the construction of the power plant dataset. We separately examined coal- or oil-fired and peaker plants (that is, plants that turn on only during high-demand days) because they typically have higher emissions rates per unit of energy produced than baseload-generating natural gas plants. All plants that came online in or after 1940 are included in our analysis of power plant siting, including those that have since been retired.

We additionally considered cumulative annual emissions from all upwind power plants within 5 km (emissions burden). Because our primary interest is in how red-lining influenced siting, we did not attempt to estimate pollution dispersion or population exposures to ambient concentrations of these pollutants. Rather, we chose the metric of emissions burden because it reflects both the number of upwind power plants and their ‘dirtiness’ (for example, generation capacity, technology, fuel type and emissions control measures, which all influence the magnitude of emissions). Because historical emissions data were not available for all time periods, we focused the emissions analysis only on plants still operable in 2019.

Because neighbourhoods graded A and D already differed substantially at the time HOLC grades were assigned, we took the approach of estimating differences associated with adjacent grades that were more similar to each other at baseline (that is, D versus C, C versus B and B versus A)^16^. We controlled for US census region and the presence of power plants before 1940. In a sensitivity analysis, we additionally controlled for 1940 population demographic and socioeconomic characteristics to better isolate the effect of red-lining independent of neighbourhood differences that pre-dated the HOLC maps.

## Power plant siting and HOLC grade

**Fig. 1 |:**
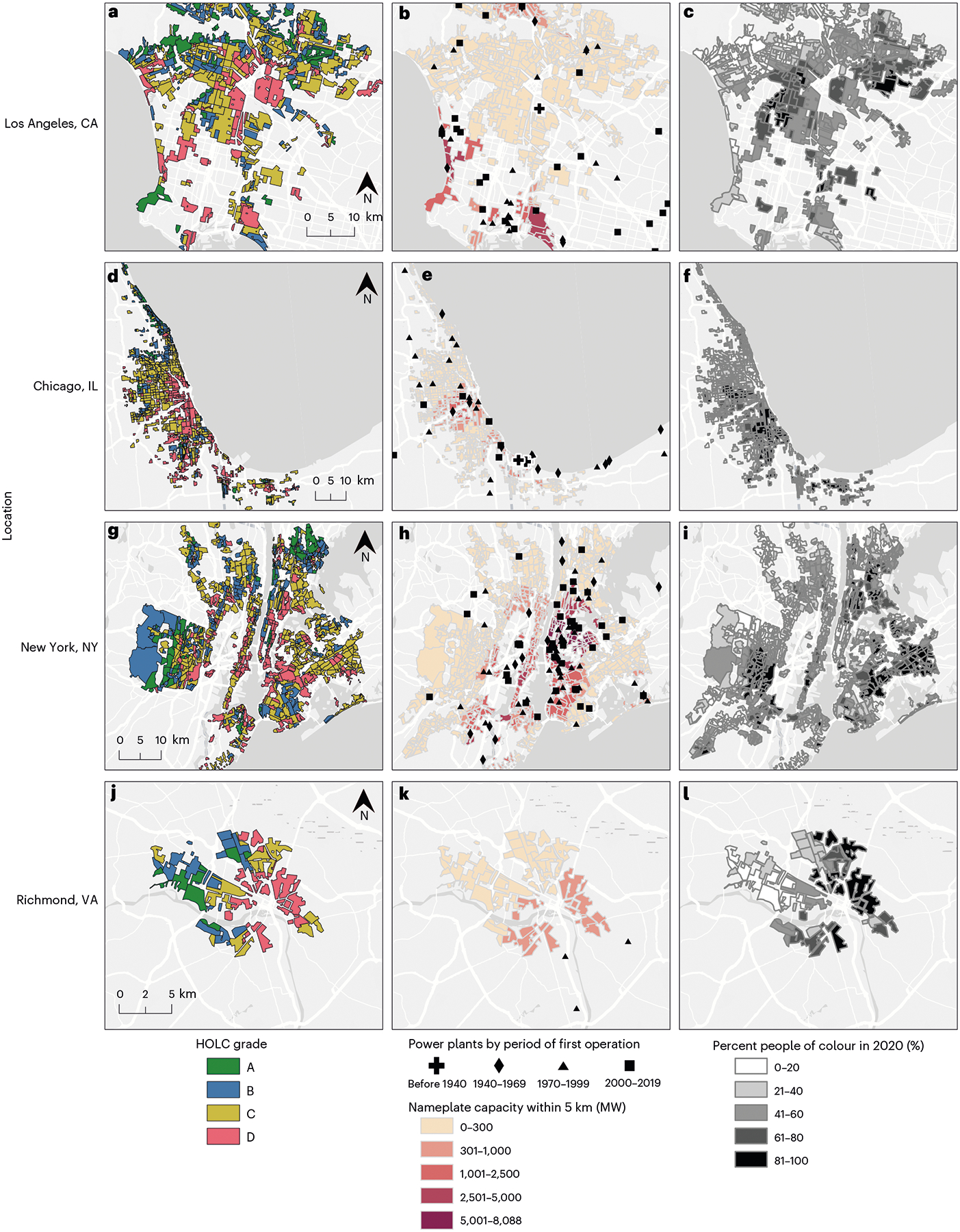
HOLC grade, fossil fuel power plant locations and neighbourhood racial/ethnic composition. **a**–**l**, Maps of Los Angeles, CA (**a**–**c**), Chicago, IL (**d**–**f**), New York, NY (**g**–**i**), and Richmond, VA (**j**–**l**) show HOLC grade (**a**,**d**,**g**,**j**), the location of fossil fuel power plants and total nameplate capacity of plants within 5 km of each HOLC neighbourhood in MW (**b**,**e**,**h**,**k**) and racial/ethnic composition in 2020 (**c**,**f**,**i**,**l**). Map data © OpenStreetMap contributors, map layer by Esri.

**Fig. 2 |:**
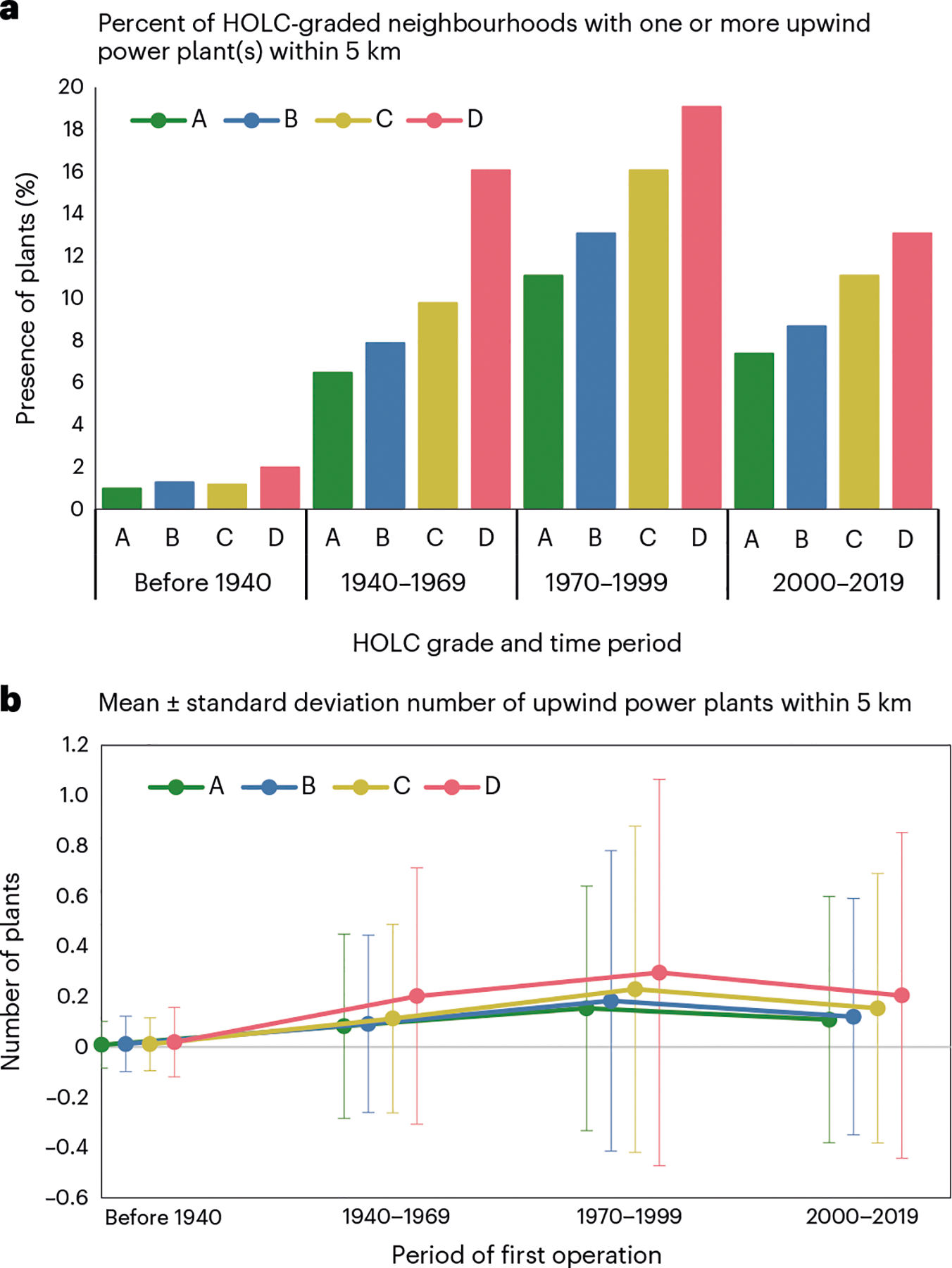
Upwind fossil fuel power plants sited within 5 km by historical redlining grade. **a**, The percent of HOLC-graded neighbourhoods with one or more upwind plant within 5 km. **b**, The mean (± standard deviation) number of upwind plants within 5 km across *n* = 8,871 HOLC-graded neighbourhoods by period of first operation.

HOLC grade, power plant locations and present-day racial/ethnic composition from the US 2020 Census are illustrated across four cities in Fig. 1. On the basis of the availability of digitized HOLC maps, we include 8,871 HOLC-graded areas (hereafter ‘neighbourhoods’) from 196 urban areas and 3,284 fossil fuel power plants in our final analysis (Table 1). Compared with other grades, a slightly higher proportion of D-graded neighbourhoods (1.9% versus 1.1%, 1.2% and 0.9% for C, B and A, respectively) already had an upwind power plant within 5 km before 1940 (Fig. 2a). The disparity grew as additional power plants came online, with the proportion of neighbourhoods with a plant nearby and average number of upwind power plants within 5 km increasing from HOLC grade A to D during subsequent periods (Fig. 2a,b).

**Fig. 3 |:**
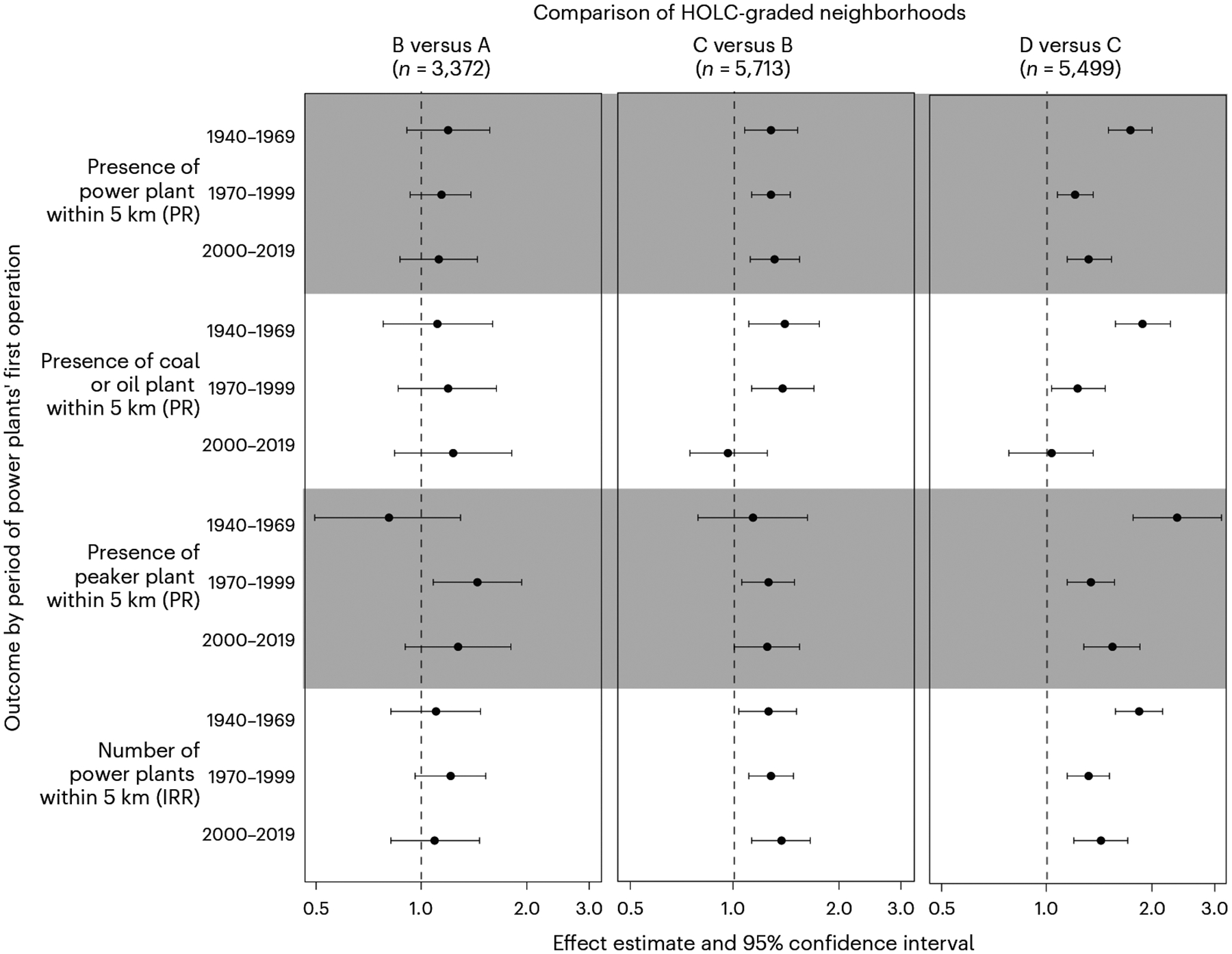
Association between historical red-lining grade and the likelihood of a fossil fuel power plant being sited upwind. Points are adjusted effect estimates from regression models controlling for the presence of power plants before 1940 and region and stratified by period of the power plants’ first operation. Error bars correspond to 95% CIs. Models include all HOLC-graded neighbourhoods. *X* axes are log scaled. PR is prevalence ratio from Poisson regression models; IRR is incidence rate ratio from negative binomial models. Dotted lines denote the null of no association. Grey shading distinguishes the outcomes.

In multivariable models comparing D-graded to C-graded areas and controlling for the presence of power plants before 1940 and US census region, red-lining was associated with a higher risk of having a fossil fuel power plant sited within 5 km during the 1940–1969 (72%), 1970–1999 (20%) and 2000–2019 (31%) periods, (prevalence ratios (95% confidence interval (CI)) = 1.72 (1.49, 1.98), 1.20 (1.07, 1.35) and 1.31 (1.14, 1.52), respectively) (Fig. 3 and Supplementary Table 2). The association was generally stronger when considering only coal or oil plants (86%, 22% and 3% higher risk over the three periods) or peaker plants (133%, 33% and 53% higher risk over the three periods) (Fig. 3 and Supplementary Tables 3 and 4). D grade was also associated with a higher number of upwind fossil fuel plants sited within 5 km, with a decrease in the association observed over time (incidence rate ratio (95% CI) = 1.82 (1.56, 2.12), 1.31 (1.14, 1.50) and 1.42 (1.19, 1.69) across the three periods, respectively) (Fig. 3 and Supplementary Table 5).

We also observed increased risks of power plant siting when comparing other adjacent grades (C versus B and B versus A) (Fig. 3 and Supplementary Tables 2–5). In general, effect estimates were smaller in magnitude and less precise, and associations were less consistently observed in every period. Unlike the D versus C comparison, the largest differences in risk when comparing other adjacent grades did not generally occur during the post-war period. For example, the strongest association between HOLC grade and power plant siting when comparing C versus B was during the 2000–2019 period.

When we additionally controlled for baseline 1940 city population size (a potential predictor of demand for new power generation) and socioeconomic variables derived from data sheets collected by HOLC appraisers (a potential predictor of subsequent power plant siting), our confidence intervals widened with the reduction in sample size due to missing data for these variables (‘Model 2’ in Supplementary Tables 2–5). Effect estimates comparing D to C grades generally nevertheless suggested similar or stronger associations between red-lining and the presence, type and number of power plants sited upwind, with the exception of the 1970–1999 period for which effect estimates were weaker (Supplementary Tables 2–5). Effect estimates were consistently attenuated and less precise for C versus B and B versus A comparisons when controlling for these baseline differences (Supplementary Tables 2–5). Effect estimates were also attenuated when we used 10 km rather than 5 km to define nearby power plants (Supplementary Fig. 2). For example, when comparing D versus C, the relationship between red-lining and the presence and number of power plants upwind and within 10 km was only apparent during the 1940–1969 period, rather than all three periods as with the 5 km analysis.

## HOLC grade and present-day power plant emissions

**Fig. 4 |:**
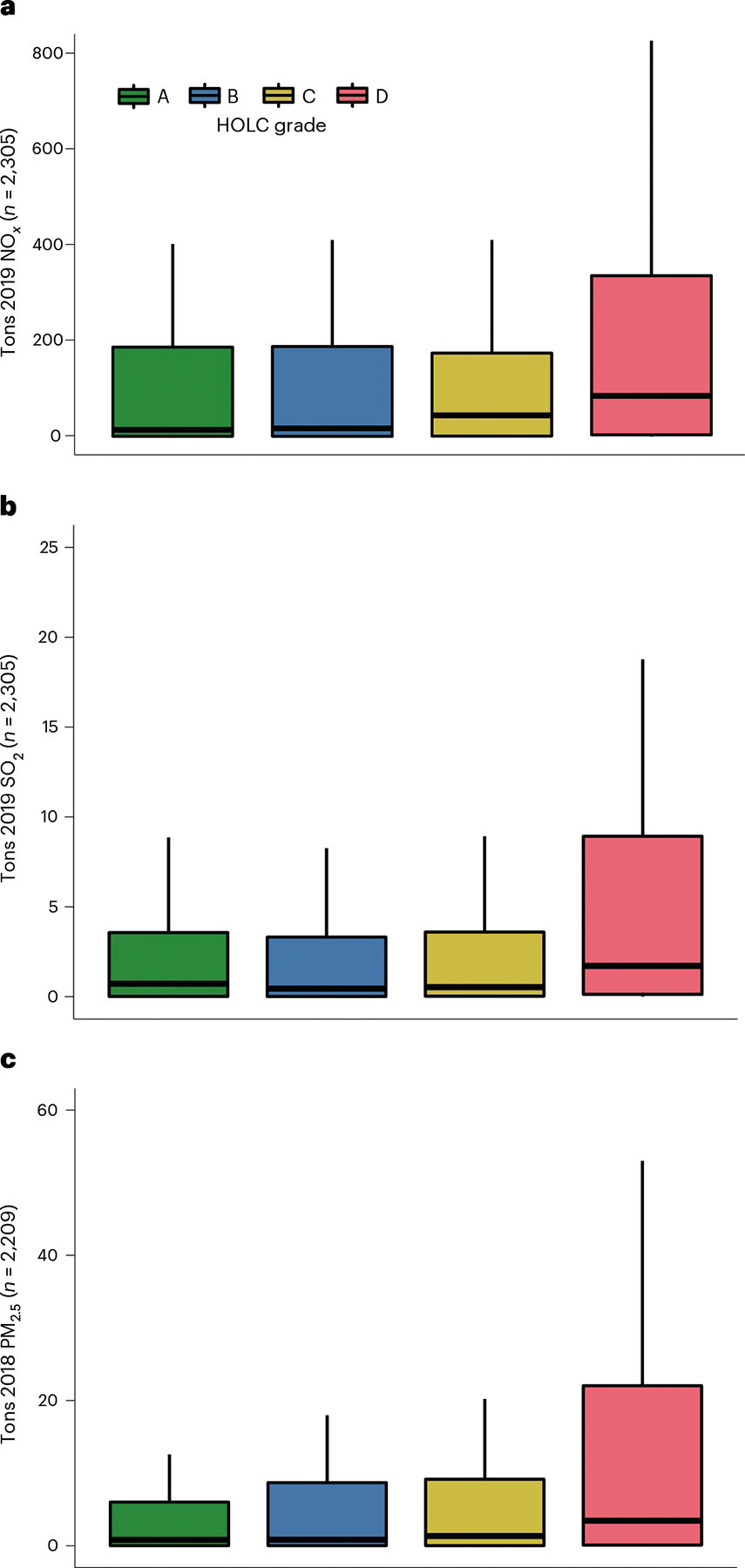
Distribution of upwind present-day power plant emissions within 5 km of HOLC-graded neighbourhoods. **a**, Tons of 2019 NO_*x*_. **b**, Tons of 2019 SO_2_. **c**, Tons of 2018 PM_2.5_. The box corresponds to the 25th percentile and 75th percentile; the middle line corresponds to the median; and the whiskers extend to the 5th and 95th percentile.

We then analysed the outcomes of present-day emissions for the 2,878 power plants still operational in 2019. We defined ‘emissions burden’ as the sum of pollutant emissions from all power plants located upwind and within a certain distance of the border of a HOLC neighbourhood; the main analysis considers a 5 km distance, while we also considered 10 km in a sensitivity analysis. Emissions burden generally followed a gradient, with A-graded neighbourhoods having the lowest on average, followed by B-, C- and D-graded neighbourhoods (Fig. 4). The trend was less pronounced for SO_2_ than it was for NO_*x*_ and PM_2.5_.

**Fig. 5 |:**
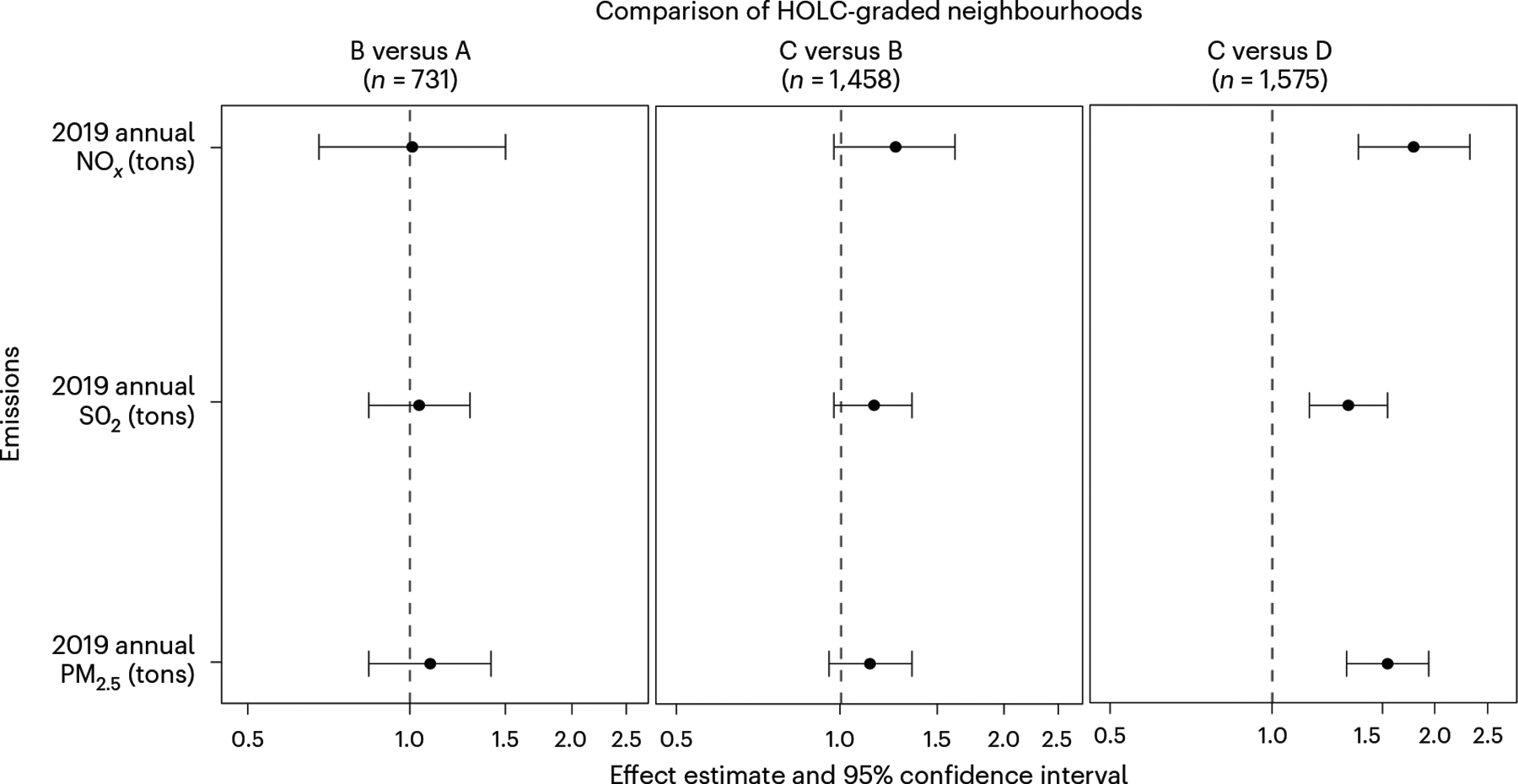
Association between historical red-lining grade and present-day power plant emissions. Points are GMRs obtained from log-linear regression models controlling for the presence of power plants before 1940 and region. Error bars correspond to 95% CIs. Models include only HOLC-graded neighbourhoods with operational upwind power plants within 5 km. *X* axes are log scaled. Dotted lines denote the null of no association.

Among neighbourhoods located downwind and within 5 km of at least one power plant, multivariate log–linear models comparing present-day emissions by grade suggested that red-lining was associated with an increase in average annual NO_*x*_ (82%), SO_2_ (38%) and PM_2.5_ (63%) emissions when comparing D- versus C-graded neighbourhoods (geometric mean ratio (GMR) and 95% CI for NO_*x*_ = ;1.82 (1.44, 2.31), for SO_2_ = 1.38 (1.17, 1.63) and for PM_2.5_ = 1.63 (1.37, 1.94)) (Fig. 5 and Supplementary Tables 6–8). A smaller positive association with emissions was also observed when comparing C- versus B-graded areas (Fig. 5 and Supplementary Tables 6–8). These associations control for the presence of a power plant before 1940 and census region.

When we additionally controlled for baseline 1940 city population size and socioeconomic characteristics, patterns remained consistent while precision decreased with the reduction in sample size due to missing data (‘Model 2’ in Supplementary Tables 6–8).The sensitivity analysis suggested a slightly (8–14%) stronger association between red-lining and emissions of all three pollutants when comparing D versus C neighbourhoods (GMR (95% CI) of 1.98 (1.32, 2.98) for NO_x_, 1.46 (1.09, 1.96) for SO_2_ and 1.86 (1.38, 2.52) for PM_2.5_; Supplementary Tables 6–8). Effect estimates were attenuated for C versus B and B versus A comparisons and remained statistically insignificant when controlling for population size and demographics. Effect estimates were also attenuated in a separate sensitivity analysis utilizing a 10 km rather than 5 km distance to define upwind power plants, while overarching patterns remained consistent (Supplementary Fig. 3).

## Discussion

Our findings suggest racism in the residential housing market as reflected in red-lining maps created by the US federal government in the 1930s had an enduring impact on the subsequent siting of fossil fuel power plants and distribution of present-day power plant emissions. Worse-graded neighbourhoods had increased risk of a fossil fuel plant being sited upwind and within 5 km, with the strongest associations between D- (‘hazardous’ or ‘red-lined’) to C-graded (‘declining’) neighbourhoods. While associations between red-lining and plant siting were strongest during the 1940–1969 period, HOLC grades assigned in the 1930s remained significantly associated with the likelihood of a plant being sited upwind over more than seven decades later (between 2000 and 2019). A ‘D’ grade was also associated with higher emissions burdens of NO_*x*_, SO_2_ and PM_2.5_ from fossil fuel power plants in the present day. Our findings are unlikely to be explained by pre-existing differences between neighbourhoods at the time the HOLC maps were created because we compared adjacent grades that have been shown to have been more similar socioeconomically based on 1940 census data^16^, controlled for differences in the presence of power plants at baseline and—in our sensitivity analyses—differences in baseline neighbourhood socioeconomic and demographic characteristics. Residence near a power plant has been associated with numerous adverse health outcomes, including increased emergency room visits^25^, asthma exacerbations^26^, respiratory-related hospitalizations^27^, preterm births^28,29^ and reduced fertility^30^, underscoring the public health importance of our findings.

Our analyses do not provide evidence regarding the specific mechanisms by which red-lining may have influenced power plant siting. Decisions about where to site new power plants are influenced by many factors including the cost of land, wages, available labour force, access to transportation and water, local and state regulations and community and political resistance^31^. The associations between red-lining and power plant siting that we observed may have arisen from depressed land values, reduced homeownership rates or reduced household income in neighbourhoods that received poorer scores^13,14^. Red-lining also deepened racial residential segregation and contributed to disinvestment in communities of colour^10,13^ that may have made power plant employment opportunities attractive in those communities and/or undermined the political influence of red-lined communities to effectively resist the nearby siting of a power plant that was not locally supported. Prior empirical work has shown that the presence of people of colour and in some cases socioeconomic status predicted subsequent siting of hazardous waste facilities and coal-fired power plants^32–35^. Evidence from California also shows industry and government officials were highly cognizant of public opposition and considered the demographic and socioeconomic characteristics of communities when deciding where to site waste-to-energy facilities to avoid local resistance^36,37^. Importantly, red-lining maps codified prevailing racism in the perception of what a home was worth based on who lived in it^38^. Thus the associations we observed were not necessarily driven by the maps themselves and how they were used, which has been debated^8,39^, but rather the racism deeply rooted in US society and market institutions of which the maps were a reflection. Future research is needed to understand the specific mechanisms through which this deep-seated racism shaped the geography of electricity generation and continues to influence decisions regarding the siting of pollution sources. Because mechanisms are likely to vary by city and region, such work would benefit from local historical investigations that were beyond the scope of the current analysis.

Present-day emissions burdens reflect siting decisions made decades earlier due to the long lifetime of power plants (median retirement age in our sample was 49 years) and the fact that older plants generally have higher emissions rates (Table 1). The disparities in emissions burden we observed are thus likely influenced by past siting decisions and variations in emissions due to factors such as size, fuel type, technology and emissions controls. Present-day community socioeconomic status and racial composition are related to the location and emissions of power plants generally and peaker plants specifically^7,40,41^. Peaker plants tend to emit higher rates of NO_*x*_ and other pollutants per unit energy produced compared with baseload-generating natural gas power plants^42^. We also found increased prevalence of peaker plants upwind of worse-graded neighbourhoods, with the largest disparity between grades D and C and plants sited during the 1940–1969 period (Fig. 3). Although we did not assess this in the current study, differences in emissions burdens could also be driven by recent power plant retirements. Nearly 100 plants have retired at least one coal-fired generator since 2014^7^. Emissions from electricity generation have declined substantially over the last several decades due to more stringent regulations and changing fuel prices that have spurred a transition from coal to natural gas. As a result, the racial gap in ambient concentrations of fine particulate matter attributable to power plants has narrowed significantly since 2000 (although a disparity remains)^43^. Historical inequalities in emissions burden across HOLC neighbourhoods are therefore likely to have been higher than what we observed in the present day.

Our findings are consistent with two prior national studies assessing the relationship between HOLC grade and present-day ambient air pollution. Namin et al. examined respiratory hazard and cancer risks associated with ambient air toxics using data from the 2014 EPA National Air Toxic Assessment (NATA). The authors observed a similar stepwise increase across HOLC grade, with air toxics risks being lowest in A-graded neighbourhoods and highest in D-graded neighbourhoods^18^. Lane et al. assessed year-2010 census block-level estimates of ambient NO_2_ and PM_2.5_ and similarly found a monotonic association with HOLC grade^19^. However, the pollutant sources considered in NATA and responsible for ambient NO_2_ and PM_2.5_ are diverse, whereas our study exclusively examined fossil fuel power plants. In a third study in California, Nardone et al. found limited differences in ambient PM_2.5_ levels by HOLC grade but much higher diesel PM emissions in worse-graded communities^23^. The lack of substantial differences in ambient PM_2.5_ in that analysis likely reflect the fact that PM_2.5_ is a regional air pollutant, while, as in our study, differences in emissions-specific sources were apparent.

In contrast to these previous studies, we examined emissions burden as opposed to ambient pollutant concentrations. Ambient air pollution attributable to a power plant will vary with distance from the plant, weather, stack height and other factors. In particular, the distribution of secondary PM formed from power plant emissions via chemical reactions in the atmosphere is likely to differ from that of primary PM emissions. SO_2_ and NO_*x*_ can both react with ammonia to create secondary PM. We also did not examine whether the differences in emissions burden that we observed were meaningful in an absolute sense for community health. It is possible that although we found HOLC grade-related differences in emissions burdens, the true health impacts of emissions may follow a different spatial pattern, including that the air quality impacts are experienced much farther away than the distances we considered in our analysis^44^. Our findings should therefore be interpreted not as measures of exposure or health impact but rather magnitude of power plant emissions as driven by the number, type and size of power plants and stringency of emissions controls.

Strengths of this study include its longitudinal nature and national scope. However, we were unable to fully isolate the causal relationship between red-lining and power plant siting given the lack of data on all possible confounders. We did control for the presence of power plants already in operation or likely in the planning stages before the creation of the HOLC maps and, in sensitivity analyses, 1940 population characteristics that may have influenced power plant siting above and beyond red-lining. This decreases, but does not rule out, the possibility that the associations we observed could have been caused by pre-existing differences between HOLC-graded areas in conditions or trends. Although we controlled for the presence of pre-existing power plants, we did not have information on other types of industry present in the 1930s. Prior work suggests the presence of industry or industrially zoned areas influenced HOLC appraisals^45,46^. It is therefore possible that correlations between already-industrialized land and worse HOLC grades in the 1930s may partially explain our results. In addition, other forms of racism in the housing market including racially restrictive covenants, predatory lending, block busting, gentrification and government programs such as Urban Renewal and construction of the Interstate Highway System also reinforced segregation^47,48^ and are likely to have shaped the geographic distribution of power generation in ways that we were not able to examine in the present analysis. We also did not assess the siting of other infrastructure projects that may be equally or more consequential for air quality and health than power plants. Construction of the Interstate Highway System in the 1950s, for example, has been shown to have contributed to further racial residential segregation and had a discriminatory impact on communities of colour, particularly urban Black neighbourhoods^49^.

The US Energy Information Administration and US Environmental Protection Agency datasets we relied on in our analysis also contained missing data that may have resulted in misclassification of some power plants and underestimation of present-day emissions. Due to the highly skewed nature of the emissions variables, model residuals were not always normally distributed even after log transformation of the outcome variables. Finally, we did not look at the racial or ethnic composition of HOLC neighbourhoods to assess the extent to which red-lining and/or power plant siting contributed to racial/ethnic disparities in power plant proximity over time. Future research could assess this question.

In conclusion, racism as codified in historic red-lining maps in the United States appears to have contributed to disparities in proximity to fossil fuel-burning power plants over time and present-day emissions burdens. While we focus on red-lining maps produced by the federal government, those maps reflected rather than created racist notions of residential property values that were widely held and deeply entrenched—indeed, capitalized upon—by the real estate industry^50^. Our findings highlight the role of racism in the residential housing market as a contributor to environmental disparities and can inform regulatory and political efforts to remedy environmental racism.

## Methods

### HOLC Security Maps

Digitized HOLC neighbourhood boundaries were downloaded as shapefiles from the University of Richmond’s Mapping Inequality Project on 20 August 2021^51^. We excluded a small number of E-graded neighbourhoods (*n* = 4) from further analysis because E was an uncommon grade.

### Power plants

Power plants are made up of one or more generators. We constructed our power plant dataset from generator-level information on operable and retired generators and plant-level locations from the 2019 US Energy Information Administration (EIA) Form 860 (https://www.eia.gov/electricity/data/eia860/) (Supplementary Fig. 1). Plants in Alaska and Hawaii were excluded because these areas were not part of the United States at the time the HOLC Security Maps were created; plants in Puerto Rico were excluded because no HOLC maps were available for Puerto Rico.

We derived the following plant-level variables: first date of operation, date of retirement, primary fuel source and nameplate capacity from the generator-level datasets. The plant’s first date of operation was defined as the first operation date of the earliest operating generator at each plant. A plant was considered retired if all of its generators had retired and the retirement date was the date of the last generator to retire.

A plant’s primary energy source was defined as the fuel source reported for the majority of its nameplate capacity on the basis of year 2019 generator-level information (for operable plants) or retirement year (for retired plants). For example, if a plant had three generators of equal nameplate capacity and two had a primary energy source of coal and one of petroleum, the plant’s primary energy source was defined as coal. Seven plants had an equal proportion (50%) of nameplate capacity coming from two energy sources. These cases were classified in the following order of priority: coal, petroleum products, natural gas and any other energy sources.

Power plants with a primary energy source other than coal, petroleum products or ‘natural gas and other gases’ were excluded from analysis. These included plants utilizing nuclear energy or renewable fuels (for example, biomass, solar, wind, geothermal, hydroelectric). For the 1940 baseline, we included a small number of plants (*n* = 16) whose primary energy source was liquid or solid renewable (biomass) fuel because fossil fuel use was less common during this time.

We used the EIA Data Atlas to identify peaker plants in our dataset (https://atlas.eia.gov/datasets/power-plants-1). We defined peaker plants as plants that currently primarily burn oil and combustion turbines primarily burning natural gas^41^.

We excluded plants that retired before 1939 from the pre-1940 baseline dataset. For all other periods (1940–1969, 1970–1999 and 2000–2019), we included all plants first operational during the period irrespective of retirement date.

### Emissions

Data on emissions of NO_*x*_ and SO_2_ were obtained from the US Environmental Protection Agency Emissions & Generation Resource Integrated Database (eGRID) for the most recent year available at the time we conducted the analysis (2019) (https://www.epa.gov/egrid). PM emissions are not currently incorporated into eGRID releases; we used draft eGRID PM_2.5_ emissions estimates for 2018 also available from the EPA. eGRID provides unadjusted estimates and estimates adjusted to reflect non-biomass derived emissions associated with electricity generation only (and not heat production). We utilized unadjusted estimates because our interest was in the health burden of emissions rather than their biogenic or non-biogenic source or purpose. Emissions were matched to our power plant dataset using the EIA plant code available in the eGRID datasets.

### Population and socioeconomic characteristics

City population size estimates for 1940 were obtained from the Mapping Inequality Project. Baseline socioeconomic and demographic characteristics of HOLC neighbourhoods were obtained from Markley, who extracted and digitized them from HOLC area description sheets^9^. These included the following variables: percent foreign born, percent Black, median household income (in dollars), median building age (in years) and three five-category ordinal variables: occupation, repair status of properties and mortgage availability. City population was available for 8,302 (94%) of HOLC neighbourhoods in our analysis, while all socioeconomic variables were available for 6,220 (70%) of neighbourhoods across 128 cities. We elected to use information from the area description sheets rather than census data as has been used in other red-lining studies because census tract boundaries do not align with HOLC neighbourhood boundaries, and only 60 US cities had census tract boundaries assigned at the time of the 1940 census. Due to the prevalence of missing data, we included these variables only in sensitivity analyses.

### Wind direction

We obtained primary monthly forcing data for the years 1980 and 2020 from the North American Land Data Assimilation System (NLDAS-2) (https://daac.gsfc.nasa.gov). Estimates are provided in 0.125° grid spacing and available from 1979 to present. Monthly zonal (*u*) and meridional (*v*) winds were used to determine the absolute wind speed (ws) by taking the square root of the summed squared *u* and *v* components ($\sqrt{u^{2}+v^{2}}=ws$). The absolute wind speed, zonal and meridional wind components were then used to obtain the leeward angle in radians (direction the wind is blowing towards) as follows: atan2 ($\frac{v}{ws},\frac{u}{ws}$). The result was converted to degrees by multiplying by 180/π and then categorized into four cardinal directions corresponding to N (NW to NE), E (NE to SE), S (SE to SW) and W (SW to NW). We used the mode of monthly observations to define the predominant wind direction at each power plant location and define downwind HOLC polygons. Because (1) data before 1979 were not available, (2) 1980 was the midpoint of our study period (1940–2020) and (3) primary wind directions in 1980 were very consistent with year 2020, we used 1980 to define primary wind direction for all power plants in our analysis.

### Analytic strategy

Spatial data were projected using the North American Datum 1983 Contiguous USA Albers coordinate system for the purposes of calculating Euclidean distances between power plants and HOLC polygon boundaries using ArcGIS version 10.5.1 (ESRI). We generated a 5 km buffer around each power plant point location and sliced the buffer into four °90 pieces corresponding to the cardinal directions. The slice corresponding to the downwind wind direction at each power plant’s location was then selected to generate the following outcomes based on intersection between the slice and the HOLC polygon boundary: presence or absence of a plant, number of plants and sum of emissions from plants. We additionally calculated the presence of a plant whose primary energy source in 2019 was coal or oil given the higher rate of criteria air pollutant emissions associated with these power plants. We also considered the presence of a peaker plant independently because when they operate, they are disproportionately polluting. We then examined descriptive statistics for each outcome stratified by HOLC grade and period.

We next conducted a series of multivariate regression models with the unit of analysis being HOLC neighbourhood using R version 4.2.0 (https://cran.r-project.org/). We took the conservative approach of comparing adjacent neighbourhood grades rather than using one grade as a reference group to better isolate the impact of red-lining, because A- and D-graded neighbourhoods likely differed more substantially from each other than C and D grades in terms of housing age, socioeconomic status and other factors that could have predicted power plant siting and for which we had limited information. Relative risks of the presence of fossil fuel power plants, coal or oil plants and peaker plants (prevalence ratios) were estimated using Poisson models stratified by time period with robust standard errors. Negative binomial models stratified by time period were used to estimate associations with the number of plants nearby (incidence rate ratios) because of over-dispersion. We used linear regression to estimate associations between HOLC grade the continuous outcomes (NO_*x*_, SO_2_ and PM_2.5_ emissions) after first log-transforming these outcomes to improve the normality of model residuals. The analysis of present-day emissions excluded HOLC neighbourhoods without a power plant that was still operable in 2019 within 5 km.

In all models, we controlled for the presence of a power plant at baseline (before 1940) and US census region (Northeast, Midwest, South or West) as potential confounders. We then undertook a sensitivity analysis in which we additionally controlled for baseline city population size and HOLC neighbourhood socioeconomic variables for the subset of HOLC neighbourhoods for which this information was available. We hypothesized that city size may confound associations with HOLC grade because larger cities were more densely populated, possibly resulting in greater demand for electricity and more densely populated neighbourhoods were more likely to be red-lined. We thought baseline socioeconomic status and demographics might confound our associations of interest because these factors were associated with HOLC grade and may have influenced subsequent siting decisions and factors such as zoning designations.

Finally, we conducted a second sensitivity analysis utilizing 10 km rather than 5 km to define nearby upwind power plants.

### Reporting summary

Further information on research design is available in the Nature Portfolio Reporting Summary linked to this article.

## Supplementary Material

Supplementary information

**Supplementary information** The online version contains supplementary material available at https://doi.org/10.1038/s41560-022-01162-y.

## Figures and Tables

**Table 1 |:** Characteristics of power plants included in the analysis by period of first operation

Variable	Before 1940 (*n*=142)	19401969 (*n*=976)	1970–1999 (*n*=1,087)	20002019 (*n*=1,079)	All time periods (*n*=3,284)
Primary energy source in 2019 *(n)*^a^					
Coal	12	222	169	18	421
Petroleum	55	291	291	228	865
Natural Gas	59	463	627	833	1,982
Other^b^	16	0	0	0	16
Retirement year (*n*)					
1980–1989	0	1	0	0	1
1990–1999	0	5	1	0	6
2000–2009	9	58	15	5	87
2010–2019	9	178	105	20	312
Years of operation of retired plants (median (min, max))	78 (63, 90)	57 (32, 79)	30 (4, 48)	10 (0, 14)	49 (0, 90)
At least one active coal or oil generator (n)	104	667	533	263	1,567
Peaker plant (n)	53	341	517	590	1,501
Nameplate capacity (MW)^c^ (median (min, max))	1 (0, 80)	40 (0, 2,372)	48 (0, 3,564)	48 (1, 4,263)	40 (0, 4,263)
2019 NO_x_ emissions intensity (tonsMW^−1^)^d,e^ (median (min, max)	3.2 (0, 985.8)	0.4 (0, 610.3)	0.5 (0, 393.1)	0.1 (0, 454.0)	0.3 (0, 985.8)
2019 SO_2_ emissions intensity (tonsMW^−1^)^d,e^ (median (min, max))	0.2 (0.0, 1,299.1)	0.0 (0.0, 524.8)	0.02 (0.0, 1,075.4)	0.01 (0.0, 1,235.6)	0.0 (0.0, 1,299.1)
2018 PM_2,5_ emissions intensity (tonsMW^−1^)^d,e^ (median (min, max))	0.2 (0.0, 145.7)	0.04 (0.0, 43.3)	0.03 (0, 7.1)	0.02 (0.0, 8.5)	0.0 (0.0, 145.7)

a Data are from the 2019 EIA Form 860.

b Includes liquid or solid renewable (biomass) fuel.

c Nameplate capacity reflects generators that were operational during the period

d Emissions data were available only for plants still operational in 2019 (total *N* = 2,878). By period of first operations, pre-1940 *N* = 124; 1940–1969 *N* = 734, 1970–1999 *N* = 966 and 2000–2019 *N* = 2,878.

e Power plants with missing values for emissions were assumed to have had zero emissions. For each period, respectively, 20, 54, 86 and 151 plants were assumed to have zero NO_*x*_ emissions; 221, 54, 88 and 151 plants were assumed to have zero SO_2_ emissions; and 22, 55, 87 and 78 plants were assumed to have zero PM_2.5_ emissions.

## Data Availability

All data used in the analysis and to create Figs. 1–5 are available publicly from the Mapping Inequality project (HOLC polygons; https://dsl.richmond.edu/panorama/red-lining/), the US Census Bureau 2020 Census (https://data.census.gov/), US Energy Information Administration Form 860 (https://www.eia.gov/electricity/data/eia860/) and Data Atlas (https://atlas.eia.gov/datasets/power-plants-1), US Environmental Protection Agency Emissions & Generation Resource Integrated Database (eGRID) (https://www.epa.gov/egrid), North American Land Data Assimilation System monthly primary forcing data (NLDAS_FORA0125_M) (https://daac.gsfc.nasa.gov/datasets/NLDAS_FORA0125_M_002/summary?keywords=NLDAS_FORA0125_M_002) and ref.^9^.

## Code availability

Analysis code is provided on GitHub at https://github.com/sherlockli1/red-lining-and-Power-Plant.
