# Supplementary material for: Historical red-lining is associated with fossil fuel power plant siting and present-day inequalities in air pollutant emissions
Source: Nat Energy. Author manuscript; Available in PMC 2026 Mar 7. (PMC12965463; doi:10.1038/s41560-022-01162-y)

nature energy

Article https://doi.org/10.1038/s41560-022-01162-y

Historical red-lining is associated with fossil fuel power plant siting and present-day inequalities in air pollutant emissions

In the format provided by the authors and unedited

Table of Contents

Supplementary Table 1. Presence of one or more fossil fuel power plant within 5km associated with HOLC grade. 2

Supplementary Table 2. Presence of one or more upwind fossil fuel power plant within 5km associated with HOLC grade. 3

Supplementary Table 3. Presence of one or more upwind coal or oil power plant within 5km associated with HOLC grade. 6

Supplementary Table 4. Presence of one or more upwind peaker plant within 5km associated with HOLC grade 9

Supplementary Table 5. Number of upwind fossil fuel power plants within 5km associated with HOLC grade. 12

Supplementary Table 6. Annual 2019 NOx emissions from upwind power plants within 5km associated with HOLC grade. 15

Supplementary Table 7. Annual 2019 SO2 emissions from upwind power plants within 5km associated with HOLC grade. 16

Supplementary Table 8. Annual 2018 PM2.5 emissions from upwind power plants within 5km associated with HOLC grade. 17

Supplementary Figure 1. Construction of power plant dataset from 2019 EIA Form 860 18

Supplementary Figure 2. Association between historical redlining grade and the likelihood of a fossil fuel power plant being sited upwind using 10km buffer distance 19

Supplementary Figure 3. Association between historical redlining grade and present-day power plant emissions using 10km buffer distance. 20

Supplementary Table 1. Presence of one or more fossil fuel power plant within 5km associated with HOLC grade. Values are prevalence ratios (PRs) from Poisson regression models with 95% confidence intervals given in parentheses. Unlike subsequent tables, predominant wind direction is not considered when defining the outcome metric.

	D vs. C (n=5,499)			C vs. B (n=5,713)			B vs. A (n=3,327)		
	**1940-1969**	**1970-1999**	**2000-2019**	**1940-1969**	**1970-1999**	**2000-2019**	**1940-1969**	**1970-1999**	**2000-2019**
HOLC grade (ref: C)	1.35 [1.25, 1.46]	1.13 [1.05, 1.21]	1.10 [1.00, 1.20]	1.24 [1.12, 1.36]	1.10 [1.03, 1.19]	1.20 [1.09, 1.32]	1.10 [0.95, 1.27]	1.18 [1.05, 1.32]	1.11 [0.97, 1.29]
Census region (ref: Northeast)									
Midwest	0.85 [0.77, 0.93]	0.66 [0.61, 0.71]	0.44 [0.39, 0.49]	0.80 [0.72, 0.89]	0.68 [0.63, 0.74]	0.42 [0.38, 0.48]	0.84 [0.71, 0.99]	0.67 [0.59, 0.75]	0.36 [0.3, 0.43]
South	0.63 [0.56, 0.71]	0.39 [0.35, 0.45]	0.16 [0.13, 0.20]	0.68 [0.6, 0.78]	0.38 [0.33, 0.43]	0.16 [0.13, 0.21]	0.87 [0.73, 1.05]	0.33 [0.27, 0.41]	0.16 [0.12, 0.22]
West	0.65 [0.56, 0.76]	0.76 [0.68, 0.84]	0.90 [0.80, 1.00]	0.69 [0.59, 0.8]	0.80 [0.73, 0.89]	1.00 [0.9, 1.11]	0.81 [0.66, 0.99]	0.86 [0.76, 0.99]	1.12 [0.97, 1.3]
Presence of power plants before 1940 (ref: No)	1.30 [1.1, 1.53]	1.23 [1.05, 1.44]	1.16 [0.92, 1.47]	1.56 [1.28, 1.9]	1.05 [0.85, 1.29]	1.27 [0.97, 1.66]	1.89 [1.44, 2.49]	0.97 [0.68, 1.38]	1.52 [1.01, 2.3]

Supplementary Table 2. Presence of one or more upwind fossil fuel power plant within 5km associated with HOLC grade. Values are prevalence ratios (PRs) from Poisson regression models with 95% confidence intervals given in parentheses.

	1940-1969		1970-1999		2000-2019	
	D vs. C					
	Model 1 (n= 5,499)	Model 2 (n= 3,813)	Model 1 (n= 5,499)	Model 2 (n= 3,813)	Model 1 (n= 5,499)	Model 2 (n= 3,867)
HOLC grade (ref: C)	1.72 [1.49,1.98]	1.70 [1.31,2.22]	1.20 [1.07,1.35]	1.07 [0.88,1.3]	1.31 [1.14,1.52]	1.22 [0.96,1.55]
Census region (ref: Northeast)						
Midwest	0.63 [0.54,0.75]	0.76 [0.61,0.95]	0.44 [0.38,0.50]	0.36 [0.3,0.44]	0.37 [0.31,0.44]	0.20 [0.15,0.27]
South	0.50 [0.40,0.62]	0.49 [0.34,0.7]	0.29 [0.23,0.35]	0.58 [0.45,0.76]	0.15 [0.11,0.21]	0.14 [0.08,0.25]
West	0.60 [0.47,0.76]	0.44 [0.29,0.67]	0.67 [0.57,0.80]	0.85 [0.69,1.06]	0.94 [0.78,1.13]	1.10 [0.87,1.39]
Presence of power plants before 1940 (ref: No)	1.07 [0.61,1.88]	2.18 [1.2,3.96]	1.24 [0.79,1.95]	1.70 [0.92,3.14]	1.46 [0.86,2.50]	3.28 [1.74,6.19]
1940 city population size (per SD)	--	1.18 [1.08,1.28]	--	1.62 [1.5,1.74]	--	1.46 [1.31,1.63]
% Black (per SD)	--	0.96 [0.88,1.04]	--	0.95 [0.89,1.02]	--	1.09 [0.76,1.57]
% Foreign born (per SD)	--	0.98 [0.9,1.08]	--	1.06 [0.98,1.13]	--	1.11 [0.98,1.25]
Median income ($1000s)	--	1.05 [1.01,1.09]	--	1.05 [1.02,1.08]	--	1.00 [1.00,1.00]
Median building age (years)	--	1.02 [1.01,1.02]	--	1.02 [1.01,1.02]	--	1.01 [1,1.02]
Lower occupation	--	1.04 [0.94,1.15]	--	1.00 [0.93,1.07]	--	0.91 [0.84,0.99]
Poorer repair status	--	1.02 [0.92,1.14]	--	1.01 [0.94,1.09]	--	0.95 [0.85,1.06]

Lower mortgage availability	--	0.99 [0.9,1.08]	--	1.03 [0.96,1.1]	--	1.03 [0.94,1.12]
AIC	4097.1	2574.6	5058.2	3486.3	3762.3	2453.6
	**C vs. B**					
	Model 1 (n= 5,713)	Model 2 (n= 3,867)	Model 1 (n= 5,713)	Model 2 (n= 3,867)	Model 1 (n= 5,713)	Model 2 (n= 3,867)
HOLC grade (ref: B)	1.27 [1.07,1.51]	0.88 [0.63,1.23]	1.27 [1.12,1.44]	1.07 [0.87,1.32]	1.30 [1.11,1.53]	1.22 [0.96,1.55]
Census region (ref: Northeast)						
Midwest	0.62 [0.51,0.76]	0.83 [0.63,1.11]	0.48 [0.41,0.55]	0.39 [0.31,0.48]	0.34 [0.28,0.42]	0.20 [0.15,0.27]
South	0.64 [0.5,0.81]	0.58 [0.39,0.87]	0.32 [0.25,0.40]	0.65 [0.49,0.86]	0.16 [0.11,0.24]	0.14 [0.08,0.25]
West	0.60 [0.45,0.78]	0.52 [0.33,0.81]	0.70 [0.59,0.83]	0.85 [0.67,1.06]	1.02 [0.84,1.23]	1.10 [0.87,1.39]
Presence of power plants before 1940 (ref: No)	1.93 [1.11,3.35]	3.38 [1.88,6.08]	0.84 [0.44,1.61]	1.08 [0.44,2.62]	1.81 [1.00,3.29]	3.28 [1.74,6.19]
1940 city population size (per SD)	--	1.08 [0.97,1.21]	--	1.70 [1.56,1.86]	--	1.46 [1.31,1.63]
% Black (per SD)	--	0.90 [0.63,1.3]	--	1.04 [0.83,1.31]	--	1.09 [0.76,1.57]
% Foreign born (per SD)	--	0.99 [0.85,1.16]	--	1.12 [1.03,1.23]	--	1.11 [0.98,1.25]
Median income ($1000s)	--	1.00 [1.00,1.01]	--	1.00 [1.00,1.01]	--	1.00 [1.00,1.00]
Median building age (years)	--	1.02 [1.01,1.03]	--	1.01 [1.00,1.02]	--	1.01 [1.00,1.02]
Lower occupation	--	1.08 [0.97,1.21]	--	0.99 [0.92,1.07]	--	0.91 [0.84,0.99]
Poorer repair status	--	1.10 [0.96,1.26]	--	1.01 [0.93,1.10]	--	0.95 [0.85,1.06]
Lower mortgage availability	--	0.99 [0.89,1.11]	--	1.02 [0.95,1.09]	--	1.03 [0.94,1.12]

AIC	3455.4	2035.3	4844.5	3234.2	1813.2	1683.6
	**B vs. A**					
	Model 1 (n= 3,327)	Model 2 (n= 2,082)	Model 1 (n= 3,327)	Model 2 (n= 2,082)	Model 1 (n= 3,327)	Model 2 (n= 2,082)
HOLC grade (ref: A)	1.19 [0.91,1.56]	0.82 [0.53,1.28]	1.14 [0.93,1.38]	1.07 [0.8,1.44]	1.12 [0.87,1.44]	1.24 [0.9,1.7]
Census region (ref: Northeast)						
Midwest	0.62 [0.46,0.85]	0.62 [0.39,0.98]	0.55 [0.44,0.68]	0.53 [0.37,0.76]	0.26 [0.19,0.37]	0.16 [0.1,0.26]
South	0.82 [0.60,1.13]	0.73 [0.41,1.27]	0.35 [0.26,0.48]	0.94 [0.64,1.4]	0.17 [0.10,0.28]	0.08 [0.03,0.22]
West	0.68 [0.47,0.99]	0.73 [0.42,1.29]	0.82 [0.65,1.04]	1.06 [0.75,1.5]	1.00 [0.77,1.29]	1.02 [0.73,1.43]
Presence of power plants before 1940 (ref: No)	3.46 [1.93,6.23]	4.54 [1.91,10.8]	0.44 [0.11,1.66]	1.2 [0.38,3.79]	2.43 [1.12,5.26]	3.44 [1.4,8.45]
1940 city population size (per SD)	--	1.35 [1.14,1.6]	--	1.88 [1.64,2.15]	--	1.49 [1.26,1.76]
% Black (per SD)	--	0.00 [0.00,0.00]	--	2.28 [1.32,3.94]	--	1.05 [0.14,7.81]
% Foreign born (per SD)	--	1.00 [0.7,1.44]	--	0.98 [0.77,1.24]	--	1.28 [1.01,1.62]
Median income ($1000s)	--	1.01 [1,1.01]	--	1.00 [1.00,1.01]	--	1.00 [1.00,1.01]
Median building age (years)	--	1.01 [0.99,1.03]	--	1.02 [1,1.03]	--	1.01 [0.99,1.02]
Lower occupation	--	1.24 [1.01,1.52]	--	1.09 [0.93,1.27]	--	0.82 [0.69,0.98]
Poorer repair status	--	1.22 [0.95,1.58]	--	0.94 [0.76,1.18]	--	0.82 [0.62,1.08]
Lower mortgage availability	--	1.11 [0.92,1.34]	--	1.04 [0.92,1.17]	--	1.15 [1.02,1.3]
AIC	1782.5	953.7	2568.3	1516.5	1813.2	1165.8

Supplementary Table 3. Presence of one or more upwind coal or oil power plant within 5km associated with HOLC grade. Values are prevalence ratios (PRs) from Poisson regression models with 95% confidence intervals given in parentheses.

	**1940-1969**		**1970-1999**		**2000-2019**	
	**D vs. C**					
	Model 1 (n= 5,499)	Model 2 (n= 3,813)	Model 1 (n= 5,499)	Model 2 (n= 3,813)	Model 1 (n= 5,499)	Model 2 (n= 3,813)
HOLC grade (ref: C)	1.86 [1.56,2.23]	1.83 [1.31,2.56]	1.22 [1.03,1.46]	1.14 [0.84,1.55]	1.03 [0.78,1.35]	1.98 [1.26,3.1]
Census region (ref: Northeast)						
Midwest	0.58 [0.48,0.7]	0.85 [0.64,1.12]	0.34 [0.28,0.42]	0.20 [0.14,0.27]	0.29 [0.21,0.40]	0.12 [0.07,0.2]
South	0.23 [0.17,0.33]	0.33 [0.19,0.59]	0.24 [0.18,0.33]	0.68 [0.47,0.99]	0.03 [0.01,0.10]	0.03 [0.01,0.15]
West	0.47 [0.34,0.64]	0.25 [0.12,0.52]	0.09 [0.05,0.16]	0.14 [0.07,0.27]	0.62 [0.43,0.90]	0.68 [0.43,1.08]
Presence of power plants before 1940 (ref: No)	1.39 [0.76,2.54]	3.69 [1.97,6.94]	2.11 [1.25,3.59]	3.29 [1.31,8.3]	3.41 [1.76,6.59]	7.86 [3.63,17.02]
1940 city population size (per SD)	--	1.24 [1.11,1.37]		1.99 [1.76,2.26]		1.05 [0.85,1.3]
% Black (per SD)	--	0.9 [0.81,1.01]	--	0.89 [0.79,1.01]	--	0.88 [0.67,1.16]
% Foreign born (per SD)	--	0.99 [0.89,1.12]	--	1.08 [0.98,1.19]	--	1.01 [0.84,1.21]
Median income ($1000s)	--	1.05 [1.02,1.09]	--	1.05 [1.02,1.08]	--	1.07 [1.03,1.11]
Median building age (years)	--	1.03 [1.02,1.03]	--	1.01 [1.01,1.02]	--	0.99 [0.97,1]
Lower occupation	--	1.09 [0.95,1.25]	--	0.92 [0.83,1.01]	--	0.90 [0.77,1.06]
Poorer repair status	--	0.96 [0.84,1.11]	--	1.03 [0.92,1.15]	--	0.79 [0.67,0.93]
Lower mortgage availability	--	1.05 [0.93,1.19]	--	1.10 [0.99,1.22]	--	1.14 [0.98,1.32]

AIC	2982.6	1770.0	2908.6	1974.3	1659.8	1129.5
	**C vs. B**					
	Model 1 (n= 5,713)	Model 2 (n= 3,867)	Model 1 (n= 5,713)	Model 2 (n= 3,867)	Model 1 (n= 5,713)	Model 2 (n= 3,867)
HOLC grade (ref: B)	1.39 [1.10,1.74]	0.87 [0.54,1.4]	1.37 [1.12,1.68]	1.18 [0.85,1.65]	0.96 [0.7,1.24]	1.25 [0.84,1.85]
Census region (ref: Northeast)						
Midwest	0.53 [0.42,0.68]	1.02 [0.71,1.48]	0.34 [0.27,0.42]	0.20 [0.13,0.31]	0.29 [0.21,0.4]	0.14 [0.08,0.23]
South	0.25 [0.17,0.38]	0.26 [0.11,0.59]	0.30 [0.22,0.40]	0.91 [0.63,1.33]	0.04 [0.02,0.12]	0.05 [0.01,0.16]
West	0.40 [0.27,0.58]	0.04 [0.00,0.53]	0.07 [0.03,0.13]	0.12 [0.06,0.26]	0.50 [0.35,0.72]	0.57 [0.37,0.87]
Presence of power plants before 1940 (ref: No)	2.42 [1.31,4.47]	6.12 [3.11,12.05]	1.13 [0.48,2.66]	0.70 [0.09,5.26]	2.85 [1.33,6.1]	4.59 [1.84,11.43]
1940 city population size (per SD)	--	1.12 [0.98,1.28]	--	1.96 [1.69,2.27]	--	1.06 [0.85,1.31]
% Black (per SD)	--	1.03 [0.57,1.86]	--	1.01 [0.74,1.4]	--	1.54 [1,2.38]
% Foreign born (per SD)	--	1.09 [0.91,1.3]	--	1.27 [1.14,1.43]	--	1.20 [1.00,1.44]
Median income ($1000s)	--	1.01 [1.00,1.02]	--	1.01 [1.00,1.01]	--	1.00 [1.00,1.01]
Median building age (years)	--	1.03 [1.02,1.04]	--	1.01 [1.00,1.02]	--	0.99 [0.98,1.01]
Lower occupation	--	1.20 [1.02,1.41]	--	0.87 [0.78,0.98]	--	0.81 [0.7,0.94]
Poorer repair status	--	0.88 [0.73,1.07]	--	1.04 [0.92,1.18]	--	0.81 [0.67,0.97]
Lower mortgage availability	--	1.16 [0.99,1.36]	--	1.13 [1.01,1.26]	--	1.06 [0.92,1.23]
AIC	2365.2	1177.6	2649.1	1713.3	1801.9	1248.0
	**B vs. A**					

	Model 1 (n= 3,327)	Model 2 (n= 2,082)	Model 1 (n= 3,327)	Model 2 (n= 2,082)	Model 1 (n= 3,327)	Model 2 (n= 2,082)
HOLC grade (ref: A) Grade B	1.11 [0.78,1.59]	0.94 [0.48,1.84]	1.19 [0.86,1.63]	1.14 [0.71,1.82]	1.23 [0.84,1.8]	1.49 [0.92,2.42]
Census region (ref: Northeast)						
Midwest	0.52 [0.36,0.75]	1.08 [0.63,1.85]	0.30 [0.21,0.43]	0.21 [0.09,0.46]	0.22 [0.15,0.35]	0.11 [0.05,0.24]
South	0.32 [0.18,0.54]	0.16 [0.04,0.69]	0.40 [0.27,0.59]	1.45 [0.89,2.35]	0.05 [0.01,0.14]	0.06 [0.01,0.23]
West	0.35 [0.20,0.62]	0.00 [0.00, 0.00]	0.03 [0.01,0.13]	0.01 [0,2.48]	0.23 [0.13,0.42]	0.27 [0.14,0.55]
Presence of power plants before 1940 (ref: No)	3.09 [1.36,7.02]	10.03 [4.06,24.75]	0.00 [0.00, 0.00]	0.00 [0.00, 0.00]	2.96 [1.05,8.34]	3.90 [1.08,14.04]
1940 city population size (per SD)	--	1.31 [1.08,1.58]	--	1.90 [1.46,2.49]	--	1.43 [1.12,1.82]
% Black (per SD)	--	0.00 [0.00,0.00]	--	2.9 [0.68,12.39]	--	0.01 [0,21.64]
% Foreign born (per SD)	--	0.97 [0.600,1.57]	--	1.22 [0.93,1.61]	--	1.15 [0.84,1.57]
Median income ($1000s)	--	1.06 [1.04,1.07]	--	1.01 [1.00,1.02]	--	1.00 [1.00,1.01]
Median building age (years)	--	1.01 [0.99,1.03]	--	1.02 [1.00,1.04]	--	1.01 [0.99,1.03]
Lower occupation	--	1.57 [1.18,2.09]	--	0.97 [0.73,1.29]	--	0.74 [0.56,0.97]
Poorer repair status	--	0.91 [0.56,1.47]	--	0.96 [0.73,1.27]	--	0.77 [0.53,1.13]
Lower mortgage availability	--	1.26 [1.02,1.57]	--	1.19 [1.02,1.4]	--	1.08 [0.90,1.31]
AIC	1173.4	488.2	1302.4	731.4	1014.9	692.0

Supplementary Table 4. Presence of one or more upwind peaker plant within 5km associated with HOLC grade.
Values are prevalence ratios (PRs) from Poisson regression models with 95% confidence intervals given in parentheses.

	1940-1969		1970-1999		2000-2019	
	D vs. C					
	Model 1 (n= 5,499)	Model 2 (n= 3,813)	Model 1 (n= 5,499)	Model 2 (n= 3,813)	Model 1 (n= 5,499)	Model 2 (n= 3,813)
HOLC grade (ref: C) Grade D	2.33 [1.75,3.11]	2.56 [1.52,4.32]	1.33 [1.14,1.55]	1.24 [0.95,1.6]	1.53 [1.27,1.83]	1.74 [1.31,2.32]
Census region (ref: Northeast)						
Midwest	0.34 [0.25,0.47]	0.29 [0.18,0.46]	0.50 [0.42,0.60]	0.33 [0.26,0.43]	0.50 [0.41,0.62]	0.28 [0.21,0.36]
South	0.28 [0.18,0.44]	0.47 [0.25,0.91]	0.39 [0.31,0.50]	0.93 [0.68,1.27]	0.11 [0.07,0.18]	0.23 [0.13,0.41]
West	0.19 [0.09,0.37]	0.00 [0.00,0.00]	0.67 [0.53,0.85]	0.60 [0.43,0.84]	0.65 [0.49,0.85]	0.51 [0.35,0.74]
Presence of Power Plants before 1940 (ref: no)	3.43 [1.76,6.66]	12.23 [5.81,25.76]	1.99 [1.26,3.14]	3.50 [1.84,6.63]	1.42 [0.75,2.70]	5.00 [2.57,9.71]
1940 city population size (per SD)	--	1.68 [1.37,2.06]	--	1.97 [1.78,2.19]	--	1.80 [1.61,2.00]
% Black (per SD)	--	0.93 [0.81,1.08]	--	0.95 [0.87,1.04]	--	0.86 [0.75,0.98]
% Foreign born (per SD)	--	0.90 [0.75,1.10]	--	1.07 [0.97,1.17]	--	0.94 [0.83,1.05]
Median income ($1000s)	--	1.1 [1.06,1.14]	--	1.07 [1.04,1.1]	--	1.04 [1.01,1.07]
Median building age (years)	--	1.03 [1.01,1.04]	--	1.02 [1.01,1.02]	--	1.01 [1.01,1.02]
Lower occupation	--	0.89 [0.74,1.08]	--	0.95 [0.87,1.05]	--	0.86 [0.77,0.96]
Poorer repair status	--	1.06 [0.86,1.30]	--	1.02 [0.93,1.13]	--	0.95 [0.84,1.07]
Lower mortgage availability	--	1.11 [0.94,1.32]	--	1.05 [0.96,1.15]	--	1.10 [0.99,1.22]
AIC	1546.7	975.1	3721.5	2496.8	2797.9	2007.9

	C vs. B					
	Model 1 (n= 5,713)	Model 2 (n= 3,867)	Model 1 (n= 5,713)	Model 2 (n= 3,867)	Model 1 (n= 5,713)	Model 2 (n= 3,867)
HOLC grade (ref: B)	1.13 [0.79,1.61]	0.91 [0.43,1.91]	1.25 [1.05,1.48]	0.93 [0.71,1.21]	1.24 [1.00,1.53]	1.42 [1.04,1.95]
Census region (ref: Northeast)						
Midwest	0.27 [0.17,0.42]	0.40 [0.21,0.75]	0.63 [0.51,0.77]	0.31 [0.22,0.42]	0.47 [0.37,0.6]	0.26 [0.19,0.36]
South	0.51 [0.32,0.81]	1.13 [0.61,2.07]	0.55 [0.43,0.71]	1.23 [0.89,1.7]	0.12 [0.07,0.21]	0.22 [0.12,0.39]
West	0.35 [0.19,0.64]	0.00 [0.00,0.00]	0.69 [0.54,0.90]	0.49 [0.34,0.72]	0.63 [0.47,0.84]	0.44 [0.3,0.65]
Presence of power plants before 1940 (ref: No)	5.06 [2.25,11.35]	13.01 [5.54,30.56]	1.40 [0.72,2.70]	2.47 [0.97,6.33]	1.53 [0.70,3.35]	3.84 [1.65,8.95]
1940 city population size (per SD)	--	1.23 [0.94,1.62]	--	2.35 [2.07,2.66]	--	1.61 [1.41,1.85]
% Black (per SD)	--	0.71 [0.25,1.97]	--	0.98 [0.72,1.33]	--	0.76 [0.33,1.73]
% Foreign born (per SD)	--	0.96 [0.67,1.38]	--	1.18 [1.05,1.32]	--	1.10 [0.95,1.29]
Median income ($1000s)	--	1.08 [1.06,1.11]	--	1.00 [1.00,1.01]	--	1.00 [1.00,1.01]
Median building age (years)	--	1.02 [1.00,1.05]	--	1.00 [0.99,1.02]	--	1.01 [1,1.02]
Lower occupation	--	0.94 [0.91,1.73]	--	0.94 [0.85,1.04]	--	0.77 [0.69,0.86]
Poorer repair status	--	1.26 [0.91,1.73]	--	1.07 [0.95,1.19]	--	1.02 [0.89,1.16]
Lower mortgage availability	--	1.21 [1.00,1.45]	--	1.06 [0.96,1.17]	--	1.05 [0.94,1.18]
AIC	1178.3	643.6	3443.0	2208.3	2487.2	1795.6
	B vs. A					
	Model 1 (n= 3,327)	Model 2 (n= 2,082)	Model 1 (n= 3,327)	Model 2 (n= 2,082)	Model 1 (n= 3,327)	Model 2 (n= 2,082)

HOLC grade (ref: A) Grade B	0.81 [0.50,1.29]	1.66 [0.56,4.97]	1.44 [1.08,1.92]	1.41 [0.95,2.09]	1.27 [0.90,1.79]	1.17 [0.77,1.79]
Census region (ref: Northeast)						
Midwest	0.32 [0.18,0.56]	0.50 [0.21,1.17]	0.83 [0.62,1.12]	0.29 [0.17,0.49]	0.37 [0.26,0.54]	0.22 [0.13,0.38]
South	0.38 [0.20,0.74]	1.26 [0.49,3.24]	0.73 [0.51,1.05]	1.74 [1.11,2.75]	0.09 [0.04,0.22]	0.13 [0.05,0.35]
West	0.40 [0.20,0.82]	0.00 [0.00,0.00]	0.85 [0.59,1.23]	0.31 [0.16,0.6]	0.62 [0.42,0.92]	0.42 [0.24,0.73]
Presence of power plants before 1940 (ref: No)	4.07 [1.31,12.64]	15.37 [3.89,60.76]	0.73 [0.19,2.85]	3.31 [0.89,12.35]	1.75 [0.56,5.44]	3.30 [0.96,11.4]
1940 city population size (per SD)	--	1.36 [0.98,1.88]	--	2.97 [2.37,3.71]	--	1.53 [1.24,1.89]
% Black (per SD)	--	0.00 [0.00,0.00]	--	1.97 [1.42,2.75]	--	1.34 [0.15,12.08]
% Foreign born (per SD)	--	0.88 [0.40,1.96]	--	0.96 [0.73,1.27]	--	1.47 [1.12,1.92]
Median income ($1000s)	--	1.07 [1.04,1.09]	--	1.01 [1.00,1.01]	--	1.00 [1.00,1.01]
Median building age (years)	--	1.00 [0.96,1.03]	--	1.01 [0.99,1.02]	--	1.01 [0.99,1.03]
Lower occupation	--	1.06 [0.66,1.7]	--	0.97 [0.79,1.18]	--	0.67 [0.52,0.86]
Poorer repair status	--	1.73 [1.09,2.75]	--	1.04 [0.79,1.39]	--	0.89 [0.63,1.26]
Lower mortgage availability	--	1.29 [0.95,1.74]	--	1.05 [0.92,1.2]	--	1.17 [1.00,1.36]
AIC	709.0	306.3	1740.3	992.0	1258.6	834.8

Supplementary Table 5. Number of upwind fossil fuel power plants within 5km associated with HOLC grade.
Values are incidence rate ratios (IRRs) from negative binomial regression models with 95% confidence intervals given in parentheses.

	1940-1969		1970-1999		2000-2019	
	D vs. C					
	Model 1 (n= 5,499)	Model 2 (n= 3,813)	Model 1 (n= 5,499)	Model 2 (n= 3,813)	Model 1 (n= 5,499)	Model 2 (n= 3,813)
HOLC grade (ref: C)	1.82 [1.56,2.12]	1.78 [1.34,2.36]	1.31 [1.14,1.50]	1.27 [1.02,1.6]	1.42 [1.19,1.69]	1.62 [1.22,2.15]
Census region (ref: Northeast)						
Midwest	0.50 [0.42,0.60]	0.61 [0.48,0.77]	0.31 [0.26,0.37]	0.25 [0.2,0.31]	0.28 [0.22,0.34]	0.14 [0.11,0.18]
South	0.42 [0.34,0.53]	0.47 [0.33,0.69]	0.31 [0.26,0.38]	0.82 [0.63,1.06]	0.1 [0.07,0.14]	0.11 [0.07,0.19]
West	0.47 [0.36,0.62]	0.44 [0.29,0.67]	0.62 [0.51,0.76]	0.81 [0.63,1.05]	0.75 [0.59,0.94]	0.96 [0.72,1.27]
Presence of power plants before 1940 (ref: No)	1.14 [0.63,2.09]	2.63 [1.33,5.19]	1.28 [0.73,2.23]	2.29 [1.12,4.66]	1.15 [0.57,2.32]	4.15 [1.97,8.75]
1940 city population size (per SD)	--	1.26 [1.13,1.39]	--	1.89 [1.72,2.07]	--	1.70 [1.52,1.91]
% Black (per SD)	--	0.95 [0.86,1.04]	--	0.91 [0.84,0.99]	--	0.86 [0.75,0.98]
% Foreign born (per SD)	--	0.97 [0.87,1.08]	--	1.03 [0.94,1.12]	--	0.97 [0.87,1.09]
Median income ($1000s)	--	1.09 [1.04,1.13]	--	1.12 [1.08,1.16]	--	1.06 [1,1.12]
Median building age (years)	--	1.02 [1.02,1.03]	--	1.01 [1.00,1.02]	--	1.01 [1,1.02]
Lower occupation	--	1.07 [0.96,1.19]	--	0.97 [0.89,1.06]	--	0.93 [0.84,1.03]
Poorer repair status	--	1.01 [0.90,1.13]	--	0.96 [0.88,1.05]	--	0.95 [0.86,1.06]
Lower mortgage availability	--	1.00 [0.91,1.10]	--	1.15 [1.07,1.24]	--	1.06 [0.96,1.16]

AIC	4700.8	2981.7	6581.7	4580.4	4832.2	3444.1
	**C vs. B**					
	Model 1 (n= 5,713)	Model 2 (n= 3,867)	Model 1 (n= 5,713)	Model 2 (n= 3,867)	Model 1 (n= 5,713)	Model 2 (n= 3,867)
HOLC grade (ref: C) Grade D	1.25 [1.03,1.50]	0.74 [0.52,1.06]	1.27 [1.10,1.47]	1.17 [0.92,1.49]	1.36 [1.12,1.64]	1.36 [1.02,1.81]
Census region (ref: Northeast)						
Midwest	0.55 [0.44,0.68]	0.77 [0.57,1.05]	0.38 [0.31,0.45]	0.30 [0.23,0.39]	0.27 [0.22,0.34]	0.14 [0.1,0.19]
South	0.57 [0.44,0.74]	0.61 [0.4,0.95]	0.45 [0.37,0.56]	1.17 [0.89,1.53]	0.11 [0.08,0.17]	0.12 [0.07,0.21]
West	0.55 [0.41,0.74]	0.68 [0.43,1.07]	0.70 [0.56,0.86]	0.85 [0.64,1.12]	0.80 [0.63,1.01]	0.97 [0.73,1.29]
Presence of power plants before 1940 (ref: No)	1.86 [0.94,3.65]	3.37 [1.39,8.14]	0.77 [0.35,1.65]	1.17 [0.42,3.27]	1.54 [0.70,3.35]	3.94 [1.7,9.15]
1940 city population size (per SD)	--	1.17 [1.02,1.33]	--	1.90 [1.71,2.11]	--	1.58 [1.38,1.79]
% Black (per SD)	--	0.9 [0.45,1.8]	--	0.85 [0.54,1.33]	--	0.84 [0.36,1.96]
% Foreign born (per SD)	--	0.96 [0.81,1.15]	--	1.16 [1.02,1.31]	--	1.10 [0.95,1.28]
Median income ($1000s)	--	1.03 [1.02,1.03]	--	1.02 [1.01,1.02]	--	1.00 [0.99,1.01]
Median building age (years)	--	1.03 [1.01,1.04]	--	1.00 [1.00,1.01]	--	1.01 [1.00,1.02]
Lower occupation	--	1.12 [1.00,1.26]	--	0.91 [0.83,0.99]	--	0.86 [0.78,0.96]
Poorer repair status	--	1.09 [0.94,1.26]	--	1.02 [0.92,1.14]	--	0.96 [0.85,1.09]
Lower mortgage availability	--	1.04 [0.93,1.17]	--	1.12 [1.03,1.21]	--	1.07 [0.97,1.18]
AIC	3899.1	2325.6	6137.9	4147.6	4387.0	3124.1
	**B vs. A**					

	Model 1 (n= 3,327)	Model 2 (n= 2,082)	Model 1 (n= 3,327)	Model 2 (n= 2,082)	Model 1 (n= 3,327)	Model 2 (n= 2,082)
HOLC grade (ref: A)	1.10 [0.82,1.47]	1.11 [0.68,1.8]	1.21 [0.96,1.52]	1.39 [0.99,1.95]	1.09 [0.82,1.46]	1.39 [0.96,2.02]
Census region (ref: Northeast)						
Midwest	0.54 [0.39,0.75]	0.74 [0.45,1.22]	0.43 [0.33,0.56]	0.45 [0.3,0.66]	0.20 [0.14,0.28]	0.13 [0.08,0.22]
South	0.63 [0.44,0.91]	0.7 [0.36,1.35]	0.44 [0.33,0.60]	1.58 [1.04,2.39]	0.12 [0.07,0.20]	0.09 [0.04,0.2]
West	0.60 [0.40,0.90]	1.03 [0.55,1.91]	0.81 [0.61,1.08]	1.04 [0.68,1.59]	0.68 [0.49,0.95]	0.9 [0.61,1.33]
Presence of power plants before 1940 (ref: No)	2.93 [1.18,7.27]	4.42 [1.19,16.43]	0.32 [0.07,1.50]	0.88 [0.16,4.93]	2.31 [0.81,6.61]	4.36 [1.44,13.23]
1940 city size per SD	--	1.40 [1.13,1.74]	--	2.17 [1.85,2.55]	--	1.57 [1.29,1.91]
% Black per SD	--	0.00 [0.00,0.00]	--	2.06 [0.36,11.99]	--	0.72 [0.03,14.97]
% foreign born per SD	--	1.00 [0.62,1.62]	--	1.01 [0.7,1.45]	--	1.42 [1.01,2]
Median income per $1000	--	1.04 [1.03,1.05]	--	1.03 [1.02,1.03]	--	1.02 [1.02,1.03]
Median building age	--	1.01 [0.98,1.03]	--	1.02 [1.00,1.04]	--	1.01 [0.99,1.03]
Occupation	--	1.30 [1.04,1.63]	--	0.98 [0.83,1.17]	--	0.78 [0.63,0.98]
Repair status of the properties	--	1.32 [0.98,1.80]	--	1.25 [0.99,1.57]	--	0.91 [0.69,1.21]
Mortgage availability for home buyers	--	1.18 [0.96,1.45]	--	1.02 [0.87,1.19]	--	1.17 [0.99,1.38]
AIC	2042.4	1106.7	3197.2	1905.1	2255.7	1487.1

Supplementary Table 6. Annual 2019 NOx emissions from upwind power plants within 5km associated with HOLC grade. Values are adjusted geometric mean ratios (GMRs) from linear regression models with 95% confidence intervals given in parentheses.

	D vs. C		C vs. B		B vs. A	
	Model 1 (n= 1,574)	Model 2 (n= 1,102)	Model 1 (n= 1,457)	Model 2 (n= 989)	Model 1 (n= 731)	Model 2 (n= 444)
HOLC grade (ref: Better) Worse Grade	1.82 [1.44,2.31]	1.98 [1.32,2.98]	1.26 [0.97,1.62]	1.17 [0.76,1.79]	1.01 [0.68,1.5]	1.08 [0.59,1.97]
Census region (ref: Northeast)						
Midwest	0.73 [0.55,0.96]	0.74 [0.52,1.04]	0.54 [0.4,0.73]	0.77 [0.53,1.14]	0.32 [0.21,0.49]	0.42 [0.23,0.78]
South	0.86 [0.57,1.30]	0.62 [0.34,1.12]	0.83 [0.53,1.29]	0.46 [0.25,0.82]	0.70 [0.38,1.3]	0.16 [0.07,0.35]
West	0.75 [0.53,1.06]	0.47 [0.3,0.76]	0.70 [0.50,0.98]	0.51 [0.32,0.81]	0.86 [0.54,1.37]	0.47 [0.25,0.89]
Presence of power plants before 1940 (ref: No)	3.98 [2.24,7.08]	8.19 [3.69,18.19]	5.39 [2.91,9.98]	10.71 [4.66,24.63]	7.63 [3.34,17.41]	12.67 [4.23,37.95]
1940 city population size (per SD)	--	0.87 [0.72,1.06]	--	0.66 [0.54,0.81]	--	0.61 [0.45,0.82]
% Black (per SD)	--	0.95 [0.82,1.1]	--	0.54 [0.18,1.63]	--	0.26 [0.04,1.88]
% Foreign born (per SD)	--	0.86 [0.71,1.05]	--	1.04 [0.8,1.34]	--	1.21 [0.59,2.45]
Median income ($1000s)	--	1.02 [0.94,1.12]	--	1.02 [0.97,1.07]	--	1.05 [1.02,1.08]
Median building age (years)	--	1.00 [0.99,1.02]	--	1.00 [0.99,1.02]	--	1.00 [0.97,1.03]
Lower occupation	--	1.00 [0.86,1.17]	--	0.94 [0.81,1.1]	--	0.9 [0.67,1.22]
Poorer repair status	--	0.93 [0.79,1.09]	--	0.95 [0.79,1.13]	--	1.17 [0.78,1.74]
Lower mortgage availability	--	1.18 [1.04,1.35]	--	1.27 [1.11,1.45]	--	1.29 [1.02,1.64]
R^2	0.03	0.07	0.03	0.09	0.06	0.12

Supplementary Table 7. Annual 2019 SO2 emissions from upwind power plants within 5km associated with HOLC grade. Values are adjusted geometric mean ratios (GMRs) from linear regression models with 95% confidence intervals given in parentheses.

	D vs. C		C vs. B		B vs. A	
	Model 1 (n= 1,574)	Model 2 (n= 1,102)	Model 1 (n= 1,457)	Model 2 (n= 989)	Model 1 (n=731)	Model 2 (n= 444)
HOLC grade (ref: Better) Worse Grade	1.38 [1.17,1.63]	1.46 [1.09,1.96]	1.15 [0.97,1.35]	1.14 [0.86,1.52]	1.04 [0.84,1.29]	1.29 [0.93,1.78]
Census region (ref: Northeast)						
Midwest	1.15 [0.95,1.4]	1.31 [1.02,1.68]	1.10 [0.91,1.34]	1.49 [1.15,1.94]	0.75 [0.59,0.95]	0.89 [0.64,1.24]
South	0.82 [0.61,1.09]	1.04 [0.68,1.59]	0.87 [0.65,1.16]	0.97 [0.65,1.44]	1.00 [0.71,1.39]	0.68 [0.44,1.05]
West	0.56 [0.44,0.72]	0.70 [0.50,0.98]	0.62 [0.50,0.78]	0.88 [0.65,1.21]	0.65 [0.50,0.83]	0.75 [0.54,1.06]
Presence of power plants before 1940 (ref: No)	0.79 [0.53,1.18]	0.85 [0.48,1.51]	0.89 [0.59,1.33]	0.84 [0.48,1.46]	1.16 [0.74,1.82]	1.08 [0.60,1.95]
1940 city population size (per SD)	--	1.16 [1.01,1.33]	--	0.92 [0.81,1.06]	--	0.88 [0.75,1.04]
% Black (per SD)	--	0.99 [0.89,1.1]	--	0.85 [0.41,1.78]	--	0.63 [0.22,1.81]
% Foreign born (per SD)	--	1.08 [0.94,1.24]	--	1.19 [1.00,1.41]	--	0.89 [0.61,1.30]
Median income ($1000s)	--	1.05 [0.98,1.12]	--	1.05 [1.02,1.08]	--	1.04 [1.03,1.06]
Median building age (years)	--	0.99 [0.98,1.00]	--	0.99 [0.98,1.00]	--	1.00 [0.98,1.01]
Lower occupation	--	1.05 [0.94,1.17]	--	1.01 [0.91,1.12]	--	0.98 [0.83,1.15]
Poorer repair status	--	0.99 [0.89,1.12]	--	0.99 [0.88,1.12]	--	1.03 [0.83,1.28]
Lower mortgage availability	--	1.09 [0.99,1.20]	--	1.14 [1.04,1.25]	--	1.10 [0.97,1.25]
R^2	0.03	0.05	0.02	0.04	0.02	0.10

Supplementary Table 8. Annual 2018 PM2.5 emissions from upwind power plants within 5km associated with HOLC grade. Values are adjusted geometric mean ratios (GMRs) from linear regression models with 95% confidence intervals given in parentheses.

	**D vs. C**		**C vs. B**		**B vs. A**	
	Model 1 (n= 1,509)	Model 2 (n= 1,049)	Model 1 (n= 1,390)	Model 2 (n= 942)	Model 1 (n= 700)	Model 2 (n= 430)
HOLC grade (ref: Better)	1.63 [1.37,1.94]	1.86 [1.38,2.52]	1.13 [0.95,1.35]	1.02 [0.76,1.37]	1.09 [0.84,1.41]	1.07 [0.72,1.61]
Census region (ref: Northeast)						
Midwest	0.42 [0.34,0.51]	0.35 [0.27,0.45]	0.45 [0.37,0.55]	0.46 [0.35,0.6]	0.39 [0.30,0.52]	0.43 [0.28,0.64]
South	0.53 [0.40,0.72]	0.32 [0.21,0.50]	0.64 [0.48,0.87]	0.36 [0.24,0.55]	0.69 [0.47,1.02]	0.27 [0.16,0.47]
West	0.43 [0.33,0.55]	0.40 [0.28,0.56]	0.50 [0.39,0.63]	0.50 [0.36,0.69]	0.55 [0.40,0.74]	0.63 [0.41,0.97]
Presence of power plants before 1940 (ref: No)	2.17 [1.44,3.27]	2.75 [1.55,4.88]	2.36 [1.57,3.56]	2.28 [1.3,4.03]	2.55 [1.51,4.31]	2.31 [1.12,4.76]
1940 city population size (per SD)	--	1.02 [0.89,1.18]	--	0.86 [0.75,0.99]	--	0.78 [0.63,0.96]
% Black (per SD)	--	1.00 [0.90,1.12]	--	0.60 [0.24,1.48]	--	0.90 [0.16,5.03]
% Foreign born (per SD)	--	0.84 [0.73,0.97]	--	0.8 [0.67,0.96]	--	0.69 [0.41,1.16]
Median income ($1000s)	--	0.98 [0.92,1.04]	--	1.01 [0.98,1.05]	--	1.04 [1.02,1.06]
Median building age (years)	--	1.01 [1.00,1.02]	--	1.00 [0.99,1.01]	--	1.01 [0.99,1.03]
Lower occupation	--	1.03 [0.92,1.16]	--	1.07 [0.97,1.2]	--	1.26 [1.03,1.54]
Poorer repair status	--	0.93 [0.83,1.05]	--	0.97 [0.86,1.1]	--	1.10 [0.83,1.45]
Lower mortgage availability	--	1.04 [0.95,1.15]	--	1.13 [1.03,1.24]	--	1.36 [1.16,1.59]
R^2	0.08	0.12	0.06	0.09	0.07	0.20

Supplementary Figure 1. Construction of power plant dataset from 2019 EIA Form 860

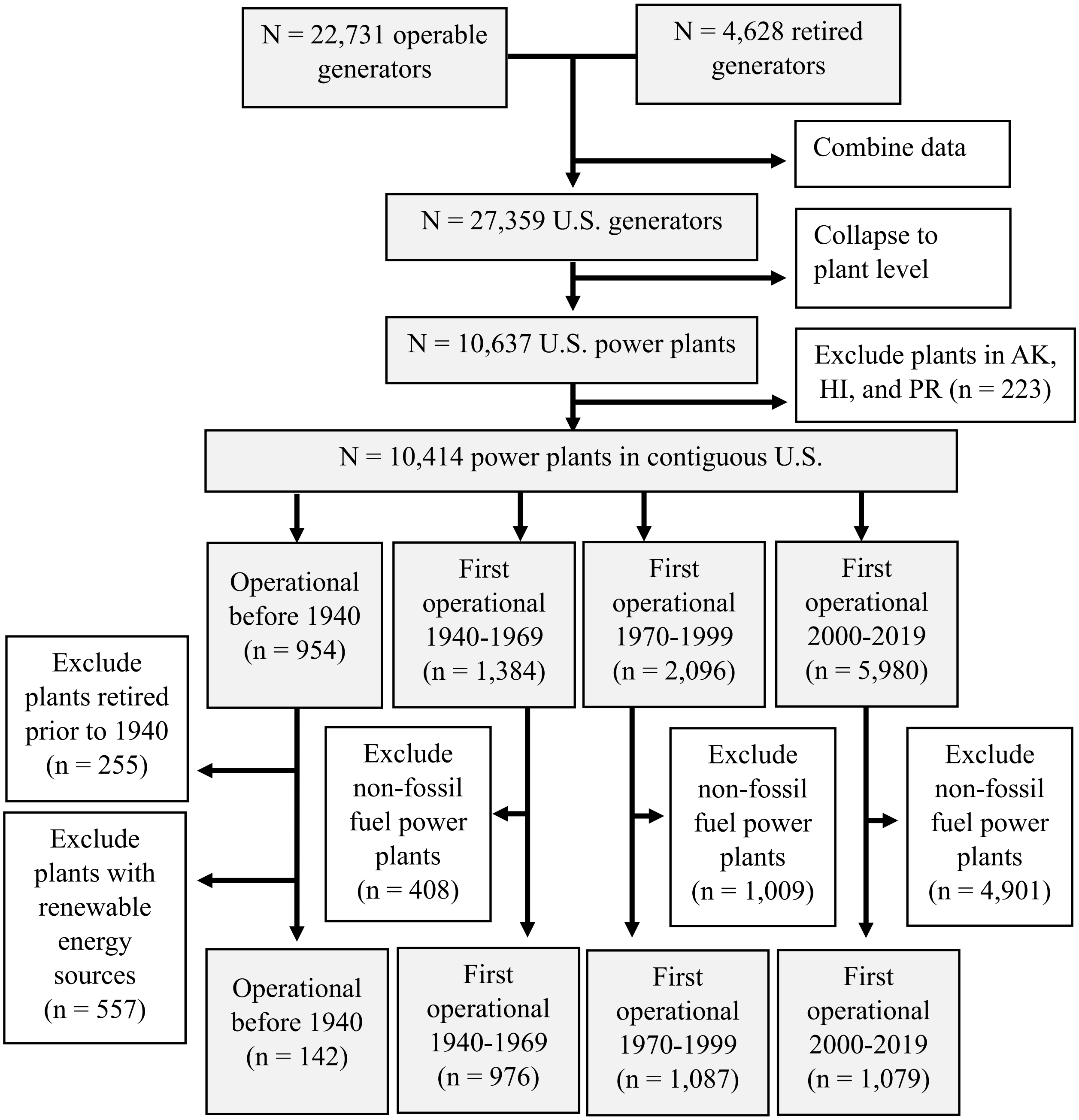

Supplementary Figure 2. Association between historical redlining grade and the likelihood of a fossil fuel power plant being sited upwind using 10km buffer distance. Points are adjusted effect estimates from regression models controlling for the presence of power plants prior to 1940 and region, and stratified by period of first operation. Error bars correspond to 95% confidence intervals (CIs). Models include all HOLC-graded neighborhoods. X-axes are log scaled. PR = prevalence ratio; IRR = incidence rate ratio.

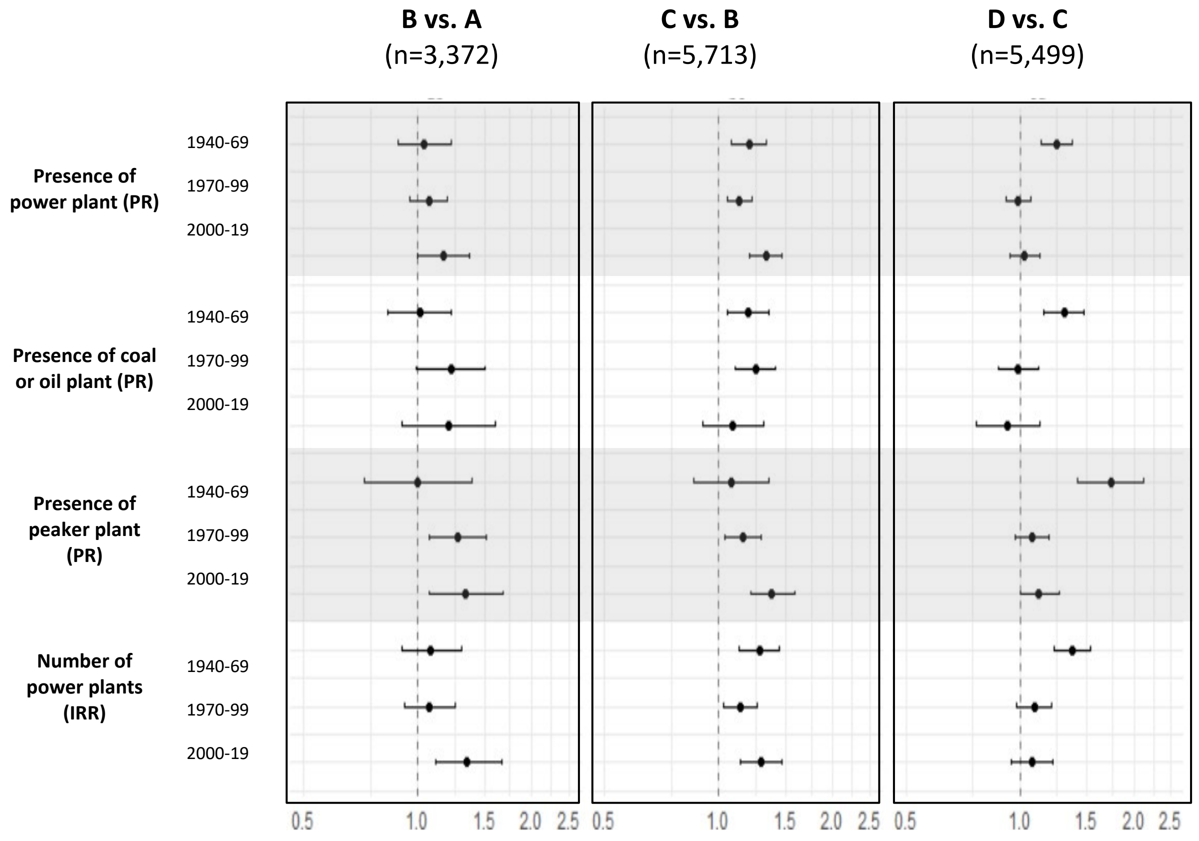

Supplementary Figure 3. Association between historical redlining grade and present-day power plant emissions using 10km buffer distance. Points are geometric mean ratios (GMRs) obtained from linear regression models controlling for the presence of power plants prior to 1940 and region. Error bars correspond to 95% confidence intervals (CIs). Models include all HOLC-graded neighborhoods with upwind power plants within 10km. X-axes are log scaled.

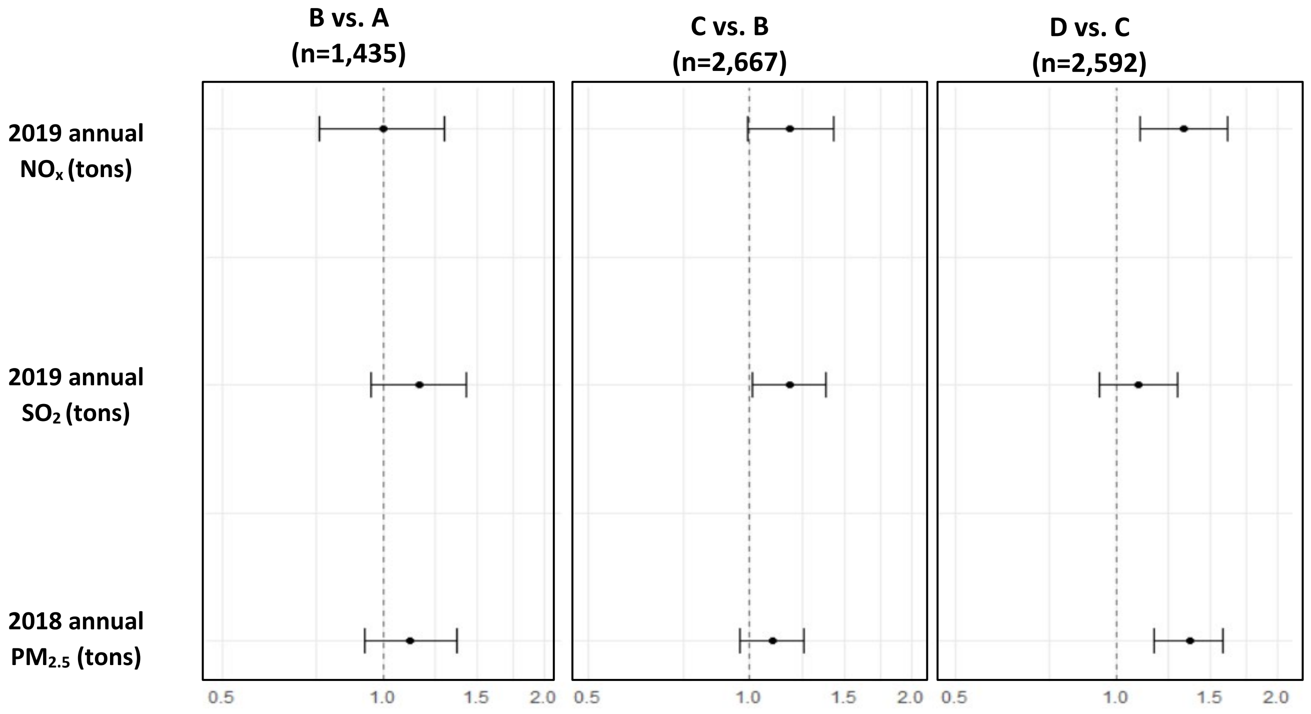

Supplement: Supplementary information [file NIHMS2151262-supplement-Supplementary_information.pdf]
